# Multi-gene phylogeny and taxonomy of *Hydnum* (*Hydnaceae*, *Cantharellales*) including a global phylogeny of ITS sequences for the genus

**DOI:** 10.3897/imafungus.17.172544

**Published:** 2026-02-03

**Authors:** Yi-Hua Xu, Long Zeng, Tai-Min Xu, Chang-Ge Song, Lu-Lu Shen, Yi-Fei Sun, Bao-Kai Cui

**Affiliations:** 1 State Key Laboratory of Efficient Production of Forest Resources, School of Ecology and Nature Conservation, Beijing Forestry University, Beijing 100083, China Beijing Forestry University Beijing China; 2 Yichang Academy of Agricultural Sciences, Yichang 443000, China Yichang Academy of Agricultural Sciences Yichang China

**Keywords:** *
Hydnum
*, molecular phylogeny, morphology, new species, species diversity

## Abstract

*Hydnum* species are called “hedgehogs” or “tooth fungi” because of their spinose hymenophores. Considering its edible and ectomycorrhizal nature, *Hydnum* has been found to possess high economic and ecological values. In the present study, a multi-gene (ITS-nLSU-*tef1α*) phylogenetic analysis and detailed morphological observations of the genus *Hydnum* were carried out. Based on the distinctive morphological characteristics and the phylogenetic evidence, two new species were described: *H. flosculoides***sp. nov**. and *H. robustum***sp. nov**. from China. Moreover, ITS is the most frequently used DNA region for identification of *Hydnum* species, but its effectiveness is uncertain. A total of 1,293 ITS sequences of *Hydnum* could be attributed to six subgenera and four clades with uncertain position (Clade *Insulana* and Incertae sedis 1, 2, 3), representing 84 morphologically and phylogenetically identified species, 14 putatively new phylospecies and 17 singletons. Furthermore, two new species were described and provided with illustrations. The morphological characters, phylogenetic delimitation, geographic distribution and habitat preferences of *Hydnum* were discussed, and the effectiveness of the ITS region was also evaluated to enhance rapid identification.

## Introduction

*Hydnum* L. (*Hydnaceae*, *Cantharellales*) was published in 1753 (Linnaeus 1753) with the type species *H. repandum* L. *Hydnum* species can establish ectomycorrhizal associations with various host plants, including *Fagaceae*, *Pinaceae*, *Malvaceae*, *Dipterocarpaceae* and *Myrtaceae*. These mutualistic associations play a crucial role in supporting forest growth and nutrient cycling within forest ecosystems (Yanaga et al. 2015; Feng et al. 2016; Cao et al. 2021; Sugawara et al. 2021; Zhang et al. 2024). *Hydnum* species are prized for their edibility, leading to their commercial sale in regions such as France, Mexico, Spain, Japan, and southwest China (Grebenc et al. 2009; He et al. 2021). Moreover, some species in this genus, such as *H. repandum* and *H. rufescens* Pers., have been confirmed to possess antioxidant and antimicrobial potential due to their extracted polyphenolic compounds (Garrab et al. 2019; Dizeci et al. 2021; He et al. 2021).

*Hydnum* species can be defined by their white to brownish-orange stipitate basidiomata with spinose hymenophore, smooth, hyaline, thin-walled, globose to ellipsoid basidiospores, and stichic, clavate to subcylindric basidia with one to eight sterigmata (Maas Geesteranus 1960; Ostrow and Beenken 2004; Olariaga et al. 2012; Vizzini et al. 2013; Yanaga et al. 2015; Feng et al. 2016; Niskanen et al. 2018; Swenie et al. 2018; Sugawara et al. 2022; Qin et al. 2024; Su et al. 2024; Zhang et al. 2024; Baroni et al. 2025; Tuo et al. 2025). Studies on the taxonomy of *Hydnum* were originally conducted using classical morphological approaches, with an initial focus on North America (Harrison 1964; Baird and Khan 1986) and Europe (Maas Geesteranus 1960; Grebenc et al. 2009; Vizzini et al. 2013). Ostrow and Beenken (2004) first utilized ITS sequences to construct a four-species unrooted phylogenetic tree of *Hydnum*, and described a new species from Germany: *H. ellipsosporum* Ostrow Beenken, characterized by its ellipsoid spores. Feng et al. (2016) identified 31 taxa of *Hydnum*, of which 26 taxa formed four distinct clades, forming the initial taxonomic system for *Hydnum*. Niskanen et al. (2018) conducted a detailed morphological and phylogenetic study of *Hydnum*, in which the number of species with molecular sequences was subsequently expanded from 12 to 49, and four subgenera were established. After that, more phylogenetic analyses were conducted on *Hydnum* species from North America (Swenie et al. 2018; Justo et al. 2023), Asia (Yanaga et al. 2015; Sugawara et al. 2022; Ali et al. 2024; Baroni et al. 2025) and Europe (Vizzini et al. 2013), discovering a total of 18 new species, and increasing the known diversity of this genus. Based on multi-gene phylogenetic analysis, Cao et al. (2021) proposed ten new species, established a new subgenus (i.e. *Brevispina*), and a new subsection (i.e. *Ovoideispora*). Following this, Zhang et al. (2024) identified seven species of *Hydnum* in China, including three novel species, i.e. *H. longipes*, *H. microcarpum*, and *H. sinorepandum*. Qin et al. (2024) further recognized six species in China with one new species (i.e. *H. erectum*). More recently, Su et al. (2024) and Tuo et al. (2025) reported eight additional species, collectively enriching the known diversity of *Hydnum* in China. Over this period, a total of 28 species were confirmed in China. As of September 2025, 978 taxa have been recorded in Index Fungorum (http://www.indexfungorum.org/) and MycoBank; however, many of them were regarded as synonyms or transferred to other genera (e.g. *Auriscalpium*, *Sarcodon*, *Hydnellum*, *Phellodon*), of which only 71 species have had their taxonomic status checked using morphological and phylogenetic evidence.

Molecular identification of *Hydnum* species often employs a multi-gene approach, utilizing markers such as the large subunit of nuclear ribosomal RNA gene (nLSU), the largest subunit of RNA polymerase II gene (*rpb1*), the second largest subunit of RNA polymerase II gene (*rpb2*), and the translation elongation factor 1-α gene (*tef1α*) (Feng et al. 2016; Niskanen et al. 2018; Swenie et al. 2018; Cao et al. 2021; Sugawara et al. 2022; Justo et al. 2023; Kim et al. 2023; Qin et al. 2024; Zhang et al. 2024). Among these, the internal transcribed spacer (ITS) region, established as the official DNA barcode for fungi, remains the most dominant marker in *Hydnum* (Summerbell et al. 2007; Schoch et al. 2012; Badotti et al. 2017). This prevalence is clearly reflected in public databases. As of September 1^st^, 2025, a total of 3,266 sequences of *Hydnum* can be retrieved from GenBank database, consisting of 1,423 ITS, 268 nLSU, 272 *tef1α*, 92 *rpb1*, 85 *rpb2*, 42 mtSSU, and eight nSSU. However, despite this abundance, the quality of available ITS sequences of *Hydnum* varies greatly, and their reliability is often questionable due to mislabelling, nomenclatural errors, incomplete or poor annotation and so on (Nilsson et al. 2006; Nilsson et al. 2012; Kõljalg et al. 2013; Badotti et al. 2017; Hofstetter et al. 2019). Therefore, a thorough examination of available ITS sequences is a prerequisite for starting phylogenetic studies in *Hydnum*.

In the light of these findings, this study aims to reconstruct an intrageneric classification framework of *Hydnum*, and revise the taxonomic status of the known species based on morphological and multi-gene (ITS-nLSU-*tef1α*) phylogenetic analyses. Furthermore, an ITS meta-analysis will be conducted to assess the effectiveness of this gene region in distinguishing various subgenera, and species within *Hydnum*, while providing accurate sequence information (i.e. sequence numbers) for each species.

## Materials and methods

### Specimen collections

All specimens examined in this study were obtained from field collections and herbarium loans. Field collections were carried out from May 2018 to April 2024 through systematic field surveys in wild forest stands. Sampling sites were distributed in France (Nancy), Australia (Tasmania), and across four provinces in China, including Sichuan (Jiuzhaigou and Kangding), Yunnan (Chuxiong, Daili, Diqing, Lanping, Mang), Hunan (Changsha) and Zhejiang (Ningbo), with elevation ranging from 800 to 3,500 m. The forest types included mixed forest, coniferous forest and broadleaf forest, dominated by *Bambusoideae*, *Fagaceae*, *Picea*, *Quercus*, *Pinus*, *Rhododendron*, *Castanopsis*, *Abies*, *Caragana*, *Betula*, *Eucalyptus* and *Nothofagus*.

A total of 29 *Hydnum* specimens were selected to served as the primary research material for this study. They were deposited in the herbarium of the Institute of Microbiology, Beijing Forestry University (BJFC), the Institute of Applied Ecology, Chinese Academy of Sciences (IFP), the Kunming Institute of Botany, Chinese Academy of Sciences (HKAS), and the Guangdong Institute of Microbiology, Guangdong Academy of Sciences (GDGM).

### Morphological studies

The habitats and macro-morphological characteristics were obtained from the field notes and laboratory observation. Microscopic characteristics were observed and illuminated from dried specimens at magnifications up to × 1000 using a Nikon Digital Sight DS-Fi2 microscope (Nikon Corporation, Tokyo, Japan). The measurements were completed with Image-Pro Plus 6.0 software (Media Cybernetics, Silver Spring, USA). The slide preparations were stained with water, 5% potassium hydroxide and Melzer’s reagent. The ultrastructures of basidia and basidiospores were photographed using a Field Emission Scanning Electron Microscope (FESEM) Hitachi SU-8010 (Hitachi, Ltd, Tokyo, Japan) at Beijing Forestry University, China (BFU). The detailed methods were following Cao et al. (2021), Sun et al. (2022), and Liu et al. (2023). Color documentation of fresh materials was following Kornerup and Wanscher (1981).

The size of basidiospores was measured on at least 30 spores per specimen. The extremes of the range (5%) were excluded and noted in parentheses. The following abbreviations were used: **IKI** = Melzer’s reagent; **IKI–** = neither amyloid nor dextrinoid; **KOH** = 5% potassium hydroxide; **av.** = average length and width of all basidiospores ± sample standard deviation; **Q** = length/width ratio of an individual basidiospore; **Qm** = average Q value of all basidiospores ± sample standard deviation; **n (a/b)** = number of spores: (a) specified quantity, (b) number of specimens.

### DNA extraction, amplification and sequencing

DNA was extracted from dried material using a CTAB rapid plant genome extraction kit-DN14 (Aidlab Biotechnologies Co., Ltd, Beijing, China). The polymerase chain reaction (PCR) was conducted following the manufacturer’s guidelines with certain modifications (Feng et al. 2016; Qin et al. 2024; Zhang et al. 2024).

The internal transcribed spacer (ITS) region of nuclear ribosomal RNA, the large subunit of nuclear ribosomal RNA gene (nLSU), and the translation elongation factor 1-α gene (*tef1α*) were used for phylogenetic analyses. The ITS and nLSU regions were amplified using primer pairs ITS5/ITS4 (White 1990) and LR0R/LR7 (Vilgalys and Hester 1990) respectively. Primer pairs HEF1F/HEF1R were employed to amplify the translation elongation factor 1-α gene (*tef1α*; Feng et al. 2016). PCRs were carried out using an S1000™ Thermal Cycler (Bio-Rad Laboratories, California, USA). The PCR procedure of ITS and *tef1α* included an initial denaturation at 95 °C for 3 min, followed by 34 cycles of denaturation at 94 °C for 40 s, annealing at 48–54 °C for 45 s and extension at 72 °C for 1 min, and a ﬁnal extension at 72 °C for 10 min. The PCR procedure of nLSU included an initial denaturation at 94 °C for 1 min, followed by 34 cycles of denaturation at 94 °C for 30 s, annealing at 50–51 °C for 1 min and extension at 72 °C for 1.5 min, and a ﬁnal extension at 72 °C for 10 min. The PCR products were puriﬁed and sequenced with the same primers at the Beijing Genomics Institute (BGI), China. All sequences analyzed in this study were deposited in GenBank and listed in Table 1.

**Table 1.** Specimen information and GenBank accession numbers of sequences used in this study.

Species name	Specimen no.	Locality	GenBank accessions			References
			ITS	nLSU	*tef1α*	
*H. aerostatisporum*	PC:0142474 (T)	USA	NR158513	—	—	GenBank
	RAS157	USA	MH379919	OR460878	—	Swenie et al. 2018
	MR00311	USA	MH379856	—	PP419923	Swenie et al. 2018; Baroni et al. 2025
*H. albertense*	HTN 11-354 (T)	CANADA	NR158492	—	—	Niskanen et al. 2018
*H. albidum*	CORT 012029 (ET)	USA	NR164025	—	PP419954	Swenie et al. 2018; Baroni et al. 2025
	AH179 (NBM)	CANADA	OQ235288	—	—	Justo et al. 2023
*H. alboaurantiacum*	RAS186 (T)	USA	MH379955	—	PP419948	Swenie et al. 2018; Baroni et al. 2025
	AJ1766 (NBM)	CANADA	OQ235292	—	—	Justo et al. 2023
*H. alboluteum*	TUMH:63988 (T)	JAPAN	NR176695	—	—	Sugawara et al. 2022
	TUMH:63992	JAPAN	LC621805	—	LC622440	Sugawara et al. 2022
*H. albopallidum*	TUM H:63997 (T)	JAPAN	NR176696	LC717904	LC622442	Sugawara et al. 2022
	TUMH:63999	JAPAN	LC621809	—	LC622444	Sugawara et al. 2022
*H. albomagnum*	TENN 073062 (ET)	USA	NR164031	—	PP419960	Swenie et al. 2018; Baroni et al. 2025
	131LL	DOMINICAN REPUBLIC	PP414163	—	PP419959	Baroni et al. 2025
*H. albomarginatum*	FJAU66574 (T)	CHINA	PV329855	PV356813	PP357262	Tuo et al. 2025
	FJAU66575	CHINA	PV329856	PV356814	PP357263	Tuo et al. 2025
*H. atlanticum*	AJ1732 (NBM) (T)	CANADA	OQ235220	—	OQ236554	Justo et al. 2023
	AJ1528 (NBM)	CANADA	OQ235211	—	—	Justo et al. 2023
*H. aurantiascens*	14LL (T)	BELIZE	PP414164	—	PP419943	Baroni et al. 2025
	MES-3176	BELIZE	ON383439	—	PP419944	Baroni et al. 2025
*H. berkeleyanum*	CAL 1656 (T)	INDIA	NR158533	NG070500	—	Wang et al. 2018
	HKAS77834	CHINA	KU612525	KU612667	—	Feng et al. 2016
	Cui 17697	CHINA	PQ505061	PQ505043	—	This study
	Cui 18728	CHINA	PQ505046	PQ505037	—	This study
	Cui 18642	CHINA	PQ505045	—	—	This study
	Dai 21255	CHINA	PQ505066	PQ505044	—	This study
*H. bifurcatum*	FJAU66562 (T)	CHINA	PV329845	—	PP357252	Tuo et al. 2025
	FJAU66563	CHINA	PV329846	—	PP357253	Tuo et al. 2025
*H. boreorepandum*	H 6003711 (T)	FINLAND	NR158488	—	—	Niskanen et al. 2018
	HTN 1679	FINLAND	KX388658	—	—	Niskanen et al. 2018
*H. brevispinum*	Wei 10214 (T)	CHINA	NR175734	MW979559	—	Cao et al. 2021
	Wei 10258	CHINA	MW980579	MW979560	—	Cao et al. 2021
*H. canadense*	HTN 09-006 (T)	CANADA	NR158495	—	—	Niskanen et al. 2018
	AJ1769 (NBM)	CANADA	OQ235164	—	—	Justo et al. 2023
	RAS100	USA	MH379892	—	PP419907	Baroni et al. 2025
*H. cf. subalpinum*	GDGM83086	CHINA	OR947118	—	—	Zhang et al. 2024
	Cui 24157	CHINA	PQ505072	—	—	This study
*H. crassipedum*	FJAU66572 (T)	CHINA	PV329853	PV356811	PP357260	Tuo et al. 2025
	FJAU66573	CHINA	PV329854	PV356812	PP357261	Tuo et al. 2025
*H. cremeum*	MHKMU TJ Yu 197 (T)	CHINA	PQ287674	PQ287755	PQ295848	Su et al. 2024
	MHKMU WH Zhang 599	CHINA	PQ287673	PQ287754	PQ295847	Su et al. 2024
*H. cremeoalbum*	TUMH:60740 (T)	JAPAN	NR176684	—	—	Yanaga et al. 2015
	HKAS92345	CHINA	KU612619	KU612676	KU612764	Feng et al. 2016
	GDGM93011	CHINA	OR947110	OR947129	—	Zhang et al. 2024
*H. crocidens*	PERTH08095981	AUSTRALIA	KU612631	KU612684	KU612797	Feng et al. 2016
	PERTH08072965	AUSTRALIA	KU612630	KU612685	—	Feng et al. 2016
*H. cuspidatum*	RAS246 (T)	USA	NR164032	—	PP419931	Swenie et al. 2018
	RAS205	USA	MH379936	—	—	Swenie et al. 2018
*H. elatum*	FRI62309	MALAYSIA	KU612637	KU612691	KU612811	Feng et al. 2016
	HKAS92352	SINGAPORE	KU612633	KU612756	KU612810	Feng et al. 2016
*H. ellipsosporum*	OS5579 (T)	GERMANY	AY817138	—	—	Ostrow and Beenken 2004
	HTN 12-036	FINLAND	KX388671	—	—	Niskanen et al. 2018
	FD3281	SWITZERLAND	KX086215	KX086217	—	GenBank
*H. erectum*	gui0024(FHMU7689) (T)	CHINA	OR722666	OR722669	—	Qin et al. 2024
*H. ferruginescens*	MH16005 (T)	USA	NR164027	—	PP419936	Swenie et al. 2018; Baroni et al. 2025
	RAS229	USA	MH379942	—	PP419937	Swenie et al. 2018; Baroni et al. 2025
*H. fibrillosum*	9543TJB (T)	COSTA RICA	PP414175	—	PP419927	Baroni et al. 2025
	AC512	PANAMA	KM594876	—	—	Baroni et al. 2025
*H. flabellatum*	Yuan 14708 (T)	CHINA	NR175732	MW979556	—	Cao et al. 2021
*H. flavidocanum*	Yuan 13903a (T)	CHINA	NR175727	MW979545	MW999440	Cao et al. 2021
	Yuan 13900a	CHINA	MW980560	MW979546	MW999441	Cao et al. 2021
*H. flavosquamosum*	MHKMU LP Tang 3454 (T)	CHINA	PQ287672	PQ287753	PQ295846	Su et al. 2024
	MHKMU LP Tang 3453	CHINA	PQ287662	PQ287744	PQ295837	Su et al. 2024
*H. flosculoides*	Cui 23128 (T)	CHINA	PQ505054	—	PX148763	This study
	Cui 23131	CHINA	PQ505055	—	PX148765	This study
	Cui 22857	CHINA	PQ505047	PQ505039	PX148768	This study
*H. formosum*	CO5236 (T)	PANAMA	PP414169	—	—	Baroni et al. 2025
	AEFM1079	COSTA RICA	PP414170	—	—	Baroni et al. 2025
*H. fulvostriatum*	FJAU66566 (T)	CHINA	PV329849	PV356807	—	Tuo et al. 2025
	FJAU66567	CHINA	PV329850	PV356808	—	Tuo et al. 2025
*H. ibericum*	BIO Fungi 12330 (T)	SPAIN	HE611086	—	—	Olariaga et al. 2012
	MA-fungi 3457	SPAIN	AJ547879	—	—	Grebenc et al. 2009
*H. itachiharitake*	TUMH:64033 (T)	JAPAN	NR176698	—	LC622462	Sugawara et al. 2022
	TUMH:64032	JAPAN	LC621829	LC717905	LC622461	Sugawara et al. 2022
*H. jussii*	H 6003709 (T)	FINLAND	NR158493	—	—	Niskanen et al. 2018
	Yuan 14008	CHINA	MW980553	MW979539	MW999436	Cao et al. 2021
	Yuan 14009	CHINA	MW980554	MW979540	MW999437	Cao et al. 2021
	Cui 18551	CHINA	PQ505063	—	PX148760	This study
*H. khanspurense*	KH09 (LAN88021) (T)	PAKISTAN	OQ130694	—	—	Ali et al. 2024
	KH50 (LAN29722)	PAKISTAN	OQ130695	—	—	Ali et al. 2024
*H. longibasidium*	Wei 10383 (T)	CHINA	NR175726	MW979541	MW999438	Cao et al. 2021
	Wei 10367	CHINA	MW980555	MW979542	MW999439	Cao et al. 2021
*H. longipes*	GDGM82458 (T)	CHINA	NR198674	—	—	Zhang et al. 2024
*H. magnorufescens*	TO HG2818 (T)	ITALY	KC293545	—	—	Vizzini et al. 2013
	161209	SLOVENIA	KU612549	KU612669	KU612795	Feng et al. 2016
*H. mcnabbianum*	PDD93278	NEW ZEALAND	KU612632	—	—	Feng et al. 2016
*H. melitosarx*	HTN 11-056 (T)	USA	NR158497	—	—	Niskanen et al. 2018
	K 176869	UK	KX388685	—	—	Niskanen et al. 2018
	GDGM84518	CHINA	OR947117	OR947136	—	Zhang et al. 2024
	Cui 18658	CHINA	—	PQ505038	—	This study
*H. melleopallidum*	UBC F17492 (T)	CANADA	NR177458	—	—	Kranabetter et al. 2009
*H. microcarpum*	GDGM879021 (T)	CHINA	NR198673	—	—	Zhang et al. 2024
	GDGM87902	CHINA	OR947116	OR947134	—	Zhang et al. 2024
	Cui 23219	CHINA	PQ505068	PQ505033	PX148769	This study
	Cui 23220	CHINA	PQ505069	PQ505034	PX148770	This study
*H. minospororufescens*	TUMH 64041 (T)	JAPAN	NR176699	—	LC622467	Sugawara et al. 2022
	TUMH 64039	JAPAN	LC621835	—	LC622466	Sugawara et al. 2022
*H. minum*	TUMH:60737 (T)	JAPAN	NR178090	—	—	Yanaga et al. 2015
	IFP 019482	CHINA	MW980557	MW979543	—	Cao et al. 2021
*H. mulsicolor*	LJU GIS 1336 (T)	SLOVENIA	NR197976	—	—	Grebenc et al. 2009
	RAS023	USA	PP414177	—	PP419911	Baroni et al. 2025
	RAS108	USA	OR464372	OR460868	—	Swenie et al. 2024
*H. neorepandum*	HTN 10-095 (T)	CANADA	NR158489	—	—	Niskanen et al. 2018
	HTN 10-086	CANADA	KX388660	—	—	Niskanen et al. 2018
*H. olympicum*	HTN 09-134 (T)	USA	NR189375	—	—	Niskanen et al. 2018
	SAT-10-208-05	USA	MT955159	—	—	GenBank
*H. oregonense*	PNW-MS g2010502h1-09 (T)	USA	NR189749	—	—	Grebenc et al. 2009
	RLP12318D	USA	OR464422	—	PP419928	Swenie et al. 2024
*H. orientalbidum*	TUMH:62998 (T)	JAPAN	—	—	LC622478	Sugawara et al. 2022
	GDGM93480	CHINA	OR947108	OR947127	—	Zhang et al. 2024
	SFC20150902-97	SOUTH KOREA	OR211381	OR211399	OR220060	Kim et al. 2023
	TUMH:64068	JAPAN	LC621862	—	LC622486	Sugawara et al. 2022
	FJAU66570	CHINA	PV329857	PV356809	PP357258	Tuo et al. 2025
	FJAU66571	CHINA	—	—	PP357259	Tuo et al. 2025
	Cui 12601	CHINA	PQ505059	—	—	This study
*H. ovoideisporum*	BIO-Fungi 12683 (T)	SPAIN	NR119818	—	—	Olariaga et al. 2012
	71106	SLOVENIA	KU612536	—	—	Feng et al. 2016
*H. pallidocroceum*	Yuan 14023 (T)	CHINA	NR175731	MW979554	MW999449	Cao et al. 2021
	Yuan 14017	CHINA	MW980569	MW979555	MW999450	Cao et al. 2021
*H. pallidomarginatum*	Yuan 13928a (T)	CHINA	NR175730	MW979552	MW999447	Cao et al. 2021
	SFC20180705-81	REPUBLIC OF KOREA	ON907792	ON907764	OR220048	Kim et al. 2023
	Cui 24163	CHINA	PQ505073	—	—	This study
*H. paucispinum*	NIBRFG0000503828 (T)	REPUBLIC OF KOREA	OR211379	OR211395	—	Kim et al. 2023
	NIBRFG0000507153	REPUBLIC OF KOREA	OR211380	OR211396	OR220063	Kim et al. 2023
	NIBRFG0000508573	REPUBLIC OF KOREA	ON907801	ON907774	OR220057	Kim et al. 2023
*H. persicinum*	44LL (T)	BELIZE	PP414172	—	PP419908	Baroni et al. 2025
	MES-3199	BELIZE	PP414173	—	PP419909	Baroni et al. 2025
*H. pinicola*	TUMH:64004 (T)	JAPAN	NR176697	—	LC622448	Sugawara et al. 2022
	TUMH:64002	JAPAN	LC621811	—	LC622446	Sugawara et al. 2022
	TUMH:64003	JAPAN	LC621812	—	LC622447	Sugawara et al. 2022
	TUMH:64000	JAPAN	LC621810	—	—	Sugawara et al. 2022
	SFC20180928-18	SOUTH KOREA	OR211383	OR211401	OR220059	Kim et al. 2023
*H. quebecense*	HTN 10-064 (T)	CANADA	NR158491	—	—	Niskanen et al. 2018
	49JD	USA	MH379876		PP419929	Swenie et al. 2018; Baroni et al. 2025
	CN9	USA	MH379881	—	—	Swenie et al. 2018
*H. reginae*	K-M 265258 (T)	UK	NR184980			Rodrigo et al. 2023
	TUF106235	ESTONIA	UDB011441	—	—	Rodrigo et al. 2023
	MA-Fungi 40149	SPAIN	AJ534975	—	—	Rodrigo et al. 2023
*H. repando-orientale*	TUMH60745 (T)	JAPAN	NR178091	—	—	Yanaga et al. 2015
	TUMH60746	JAPAN	AB906684	—	—	Yanaga et al. 2015
*H. repandum*	H 6003710 (T)	FINLAND	NR164553	—	—	Niskanen et al. 2018
	HKAS93253	GERMANY	KU612581	—	KU612769	Feng et al. 2016
	Cui 18632	FRANCE	PQ505064	—	PX148761	This study
	Cui 18633	FRANCE	PQ505065	—	PX148761	This study
*H. robustum*	Cui 22893 (T)	CHINA	PQ505050	PQ505042	PX148767	This study
	Cui 22901	CHINA	PQ505048	PQ505040	PX148766	This study
	Cui 22904	CHINA	PQ505049	PQ505041	PX148764	This study
	GDGM93020	CHINA	OR947109	OR947128	—	Zhang et al. 2024
	GDGM83047	CHINA	OR947119	OR947137	—	Zhang et al. 2024
*H. roseoalbum*	MHKMU WH Zhang 606 (T)	CHINA	PQ287669	PQ287751	PQ295844	Su et al. 2024
	MHKMU WH Zhang 606-1	CHINA	PQ287670	PQ287752	PQ295845	Su et al. 2024
*H. roseotangerinum*	MHKMU LP Tang 3458 (T)	CHINA	PQ287675	PQ287756	PQ295849	Su et al. 2024
	MHKMU LP Tang 34581	CHINA	PQ287676	PQ287757	PQ295850	Su et al. 2024
*H. rufescens*	H 6003708 (T)	FINLAND	NR173157	—	—	Niskanen et al. 2018
	HTN 7839	ESTONIA	KX388656	—	—	Niskanen et al. 2018
*H. sinorepandum*	GDGM82445 (T)	CHINA	NR198675	OR947139	—	Zhang et al. 2024
	GDGM82416	CHINA	OR947120	OR947138	—	Zhang et al. 2024
	Dai 22816	CHINA	PQ505067	—	—	This study
*H. slovenicum*	LJU GIS 1338 (T)	SLOVENIA	NR158460	—	—	Grebenc et al. 2009
*H. sphaericum*	Wei 10243 (T)	CHINA	NR175729	MW979549	MW999444	Cao et al. 2021
	Wei 10262	CHINA	MW980565	MW979551	MW999446	Cao et al. 2021
	Wei 10300	CHINA	MW980564	MW979550	MW999445	Cao et al. 2021
*H. subalpinum*	TUMH:64016 (T)	JAPAN	—	LC717891	—	Sugawara et al. 2022
	TUMH:64630	JAPAN	LC717915	LC717894	LC717876	Sugawara et al. 2022
*H. subberkeleyanum*	TUMH:64075 (T)	JAPAN	NR176700	—	LC622506	Sugawara et al. 2022
	AJ1570 (NBM)	CANADA	OQ235173	—	—	Justo et al. 2023
*H. subolympicum*	DAOM744368 (T)	CANADA	NR189375	—	—	Niskanen et al. 2018
	F1188765	USA	KU612599	KU612653	—	Feng et al. 2016
*H. subovoideisporum*	H 6003707 (T)	FINLAND	NR158494	—	—	Niskanen et al. 2018
*H. subtilior*	TENN:073034 (T)	USA	NR164029	—	PP419915	Swenie et al. 2018; Baroni et al. 2025
	Cif3425	MEXICO	PP414165	—	PP419918	Baroni et al. 2025
	TENN:073050	USA	MH379918	—	—	Swenie et al. 2018
*H. tangerinum*	IFP 019473 (T)	CHINA	NR175735	MW979561	—	Cao et al. 2021
	IFP 019474	CHINA	MW980581	MW979562	—	Cao et al. 2021
	IFP 019475	CHINA	MW980582	MW979563	—	Cao et al. 2021
*H. tenuistipitum*	Wei 10410 (T)	CHINA	NR175733	MW979557	—	Cao et al. 2021
	Wei 10417	CHINA	MW980577	MW979558	—	Cao et al. 2021
	GDGM91207	CHINA	OR947113	OR947132	—	Zhang et al. 2024
	GDGM92097	CHINA	OR947112	OR947131	—	Zhang et al. 2024
	Cui 23224	CHINA	PQ505070	PQ505035	PX148771	This study
	Cui 23231	CHINA	PQ505071	PQ505036	PX148772	This study
*H. tomaense*	TUMH:64086 (T)	JAPAN	NR176701	LC717907	LC622509	Sugawara et al. 2022
	TUMH:64085	JAPAN	LC621884	—	LC622508	Sugawara et al. 2022
*H. tottoriense*	TUMH:64091 (T)	JAPAN	NR176702	—	LC622514	Sugawara et al. 2022
	TUMH:64092	JAPAN	LC621891	—	LC622515	Sugawara et al. 2022
*H. treui*	TU(M) TU110403 (T)	PAPUA NEW GUINEA	UDB013043	—	—	Niskanen et al. 2018
	N.K. Zeng8373	CHINA	OR722667	OR722670	—	Qin et al. 2024
	(FHMU7690)					
	N.K. Zeng8374	CHINA	OR722668	OR722671	—	Qin et al. 2024
	(FHMU7691)					
*H. umbilicatum*	CORT 012241 (T)	USA	NR164026	—	PP419933	Swenie et al. 2018; Baroni et al. 2025
	UBC F28405	CANADA	KP454006	—	—	GenBank
*H. vagabundum*	CLO4985 (T)	USA	NR164028	—	—	Swenie et al. 2018
	CORT:014461	USA	MH379949	—	PP419942	Swenie et al. 2018; Baroni et al. 2025
*H. ventricosum*	Yuan 14536 (T)	CHINA	NR175728	MW979547	MW999442	Cao et al. 2021
	Yuan 14601	CHINA	MW980562	MW979548	MW999443	Cao et al. 2021
*H. vesterholtii*	BIO Fungi 10429 (T)	FRANCE	NR119819	—	—	Olariaga et al. 2012
	BIO Fungi 10452	SPAIN	HE611085	—	—	Olariaga et al. 2012
*H. washingtonianum*	WTU:014341 (T)	USA	MH379846	—	—	Swenie et al. 2018
*H. zongolicense*	MEXU 26248 (T)	MEXICO	NR177463	—	—	Niskanen et al. 2018
*Hydnum* sp.	Cui 16706	AUSTRALIA	PQ505060	—	PQ609370	This study
	BH2066F	AUSTRALIA	JF960784	—	—	GenBank
	GD1588	AUSTRALIA	KU612620	KU612688	KU612805	Feng et al. 2016
	PERTH07830742	AUSTRALIA	KU612622	KU612689	—	Feng et al. 2016
	PERTH08072957	AUSTRALIA	KU612621	—	—	Feng et al. 2016
	wi8T4spel	CHINA	KC679834	—	—	GenBank
	wi1A4spel	CHINA	KC679833	—	—	GenBank
	FRI62832	MALAYSIA	KU612625	—	—	Feng et al. 2016
	GD1584	AUSTRALIA	OR354970	—	—	Feng et al. 2016
	SL1121	SINGAPORE	KU612626	—	—	Unpublished
	PGK13_025_1	NEW CALEDONIA	KY774222	—	—	Unpublished
*Hydnum* sp.	F1187537	CANADA	KU612544	—	—	Feng et al. 2016
	HKAS92340	CHINA	KU612543	KU612661	KU612779	Feng et al. 2016
	HKAS55325	CHINA	KU612613	—	—	Feng et al. 2016
	HKAS93259	CHINA	KU612612	KU612683	KU612803	Feng et al. 2016
	HKAS92350	CHINA	KU612610	KU612672	KU612799	Feng et al. 2016
	HKAS92349	CHINA	—	KU612671	KU612798	Feng et al. 2016
	PDD93275	NEW ZEALAND	KU612624	—	KU612806	Feng et al. 2016
	PDD98029	NEW ZEALAND	KU612623	KU612690	—	Feng et al. 2016
	PDD94968	NEW ZEALAND	KU612627	—	—	Feng et al. 2016
	GD1590	AUSTRALIA	KU612629	KU612686	KU612809	Feng et al. 2016
	GD1589	AUSTRALIA	KU612628	KU612687	KU612808	Feng et al. 2016
	PERTH07608543	AUSTRALIA	KU612641	—	—	Feng et al. 2016
	PERTH08018413	AUSTRALIA	KU612642	KU612696	—	Feng et al. 2016
	PERTH08091676	AUSTRALIA	KU612640	—	—	Feng et al. 2016
	PERTH08093865	AUSTRALIA	KU612643	KU612695	KU612804	Feng et al. 2016
*Sistotrema muscicola*	taxon:154757	FINLAND	AJ606041	AJ606041	—	Nilsson et al. 2006
	KHL 11721	FINLAND	AJ606040	AJ606040	—	Nilsson et al. 2006

Note: the (T) or (ET) annotated in the table represents the holotype or epitype specimens. Newly generated sequences are in bold.

### Phylogenetic analyses

All sequences were sourced from the GenBank or UNITE databases, and the sequence selection was prioritized by type-derived materials (holotype, epitype, or ex-type) where available to ensure taxonomic accuracy. For species lacking available type sequences, the representative sequences documented in authoritative publications were adopted. All datasets used in this study were aligned in MAFFT v. 7 (Katoh and Standley 2013), and manually adjusted in BioEdit (Hall 2002). *Sistotrema muscicola* (Pers.) S. Lundell was chosen as outgroup in both multi-gene and ITS phylogenetic analyses (Cao et al. 2021).

For the multi-gene sequences dataset (ITS-nLSU-*tef1α*), a total of 426 sequences representing 232 specimens were employed in this study (227 ITS, 91 nLSU, and 108 *tef1α* sequences), including 47 newly generated sequences (22 ITS, 11 nLSU, and 14 *tef1α* sequences). The dataset was then concatenated for subsequent phylogenetic analyses. Both Maximum likelihood (ML) and Bayesian Inference (BI) analyses were conducted following the settings of Cao et al. (2021) and Song et al. (2025). ML analyses were conducted in IQ-TREE v3.0.1 using ModelFinder (MFP) to select substitution models (Nguyen et al. 2015), and HKY+F+I+R3 was selected as the best-fit model under BIC. The best-scoring ML tree had a log-likelihood of −15,202.624, and support values were obtained from 1,000 ultrafast bootstrap and 1,000 SH-aLRT replicates. BI analyses were performed using MrBayes v. 3.2.7. The analysis employed ten independent Markov Chain Monte Carlo (MCMC) chains (one cold, nine heated) and ran for nine million generations, sampling every 1,000 generations. A GTR+I+G substitution model with Dirichlet (1,1,1,1) state frequency priors were applied. Convergence was assessed by confirming the average standard deviation of split frequencies below 0.01. The ﬁrst 25% sampled trees were discarded as burn-in, while the remaining ones were used to calculate Bayesian posterior probabilities (BPP). In addition, individual gene datasets (ITS, nLSU, and *tef1α*) were also analyzed separately using the same ML and BI methodologies as described for the multi-gene dataset, with BI runs proceeding for ten million generations.

For the meta-analysis of ITS sequences, 1,439 ITS sequences were preliminarily selected, then those sequences that were shorter than 350 bases were excluded, along with erroneous genus assignments, unresolved identities, high heterogeneity or low-quality (Suppl. material 1: table SS1). The remaining 1,293 ITS sequences (including newly-generated sequences from this study) were shown in Suppl. material 1: table SS2. Then the Maximum likelihood (ML) analyses were conducted based on 1,293 ITS sequences, and the detailed methods were the same as the multi-gene analyses. ModelFinder selected TN+F+I+G4 as the best-fit model under BIC. The best ML tree had a log-likelihood of −10,173.048, with support derived from 1,000 ultrafast bootstrap and 1,000 SH-aLRT replicates.

The ML bootstrap values ≥ 75% and Bayesian posterior probabilities (BPP) ≥ 0.95 were presented on topologies from ML analyses. All trees were viewed and annotated in FigTree v. 1.4.3.

## Results

### Multi-gene phylogenetic analyses

The Bayesian Inference (BI) analysis resulted in an average standard deviation of split frequencies of 0.005716. After discarding the first 25% of sampled trees as burn-in, a total of 375,000 trees from the analysis were used to construct the majority-rule consensus tree and calculate posterior probabilities. The effective sample sizes (ESS) for all parameters were well above 200, confirming that the MCMC sampling was adequate. The alignments and phylogenetic tree topology were archived in Suppl. materials 2–4. Individual gene datasets of ITS, nLSU, and *tef1α* also converged reliably, with split frequency standard deviations of 0.004419, 0.004144, and 0.003553, respectively. The alignments and topologies of single-gene trees were provided in Suppl. materials 5–7, and the three single-gene trees with Bayesian support values annotated on the ML trees were showed in Suppl. material 8.

**Figure 1.**
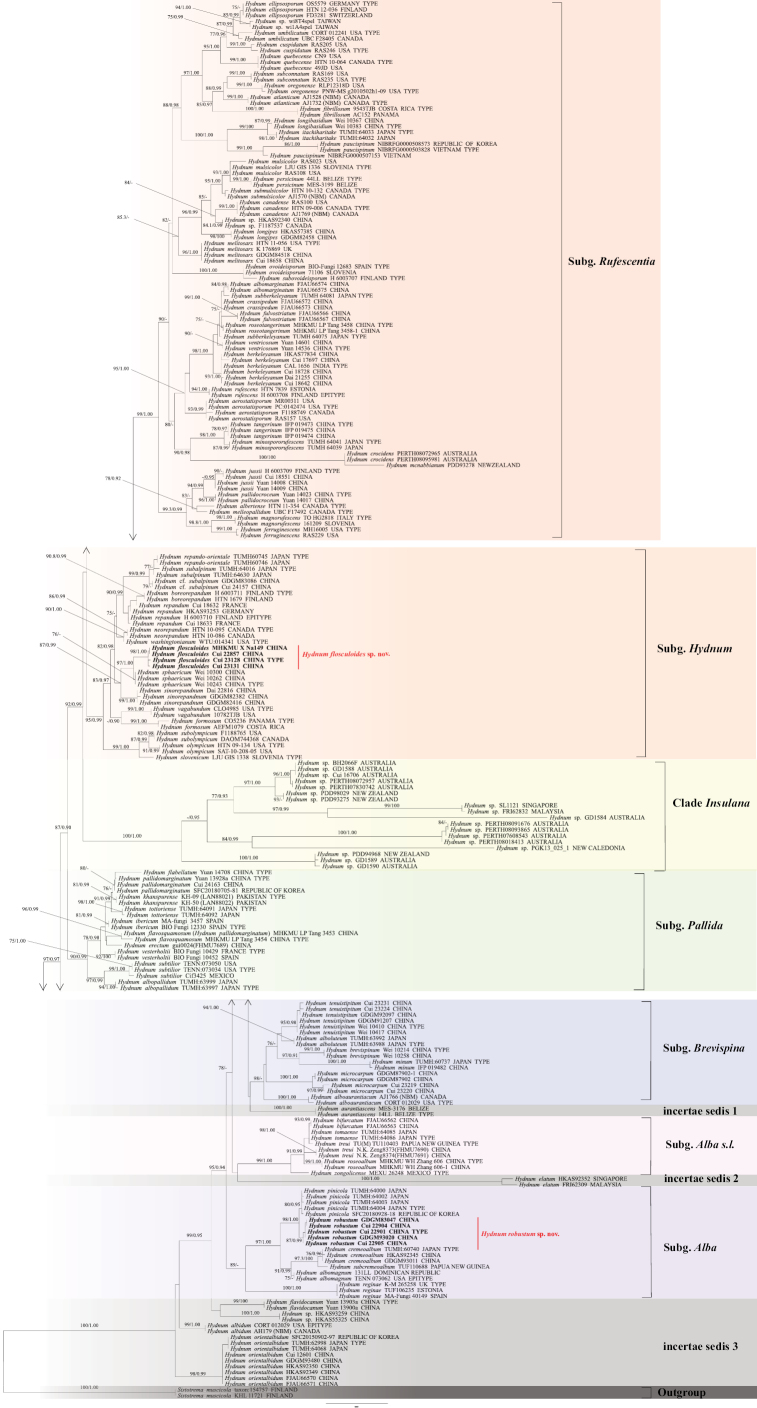
Maximum likelihood tree of the *Hydnum* based on the combined dataset (ITS-nLSU-*tef1α*). Branches are labeled with ML bootstrap values higher than 75%, and Bayesian posterior probabilities higher than 0.95 respectively. Bold names = New species.

The phylogenetic tree shows that the species of *Hydnum* are divided into six subgenera and four clades with uncertain position (Clade *Insulana* and Incertae sedis 1, 2, 3) (Fig. 1). The species composition is listed below:

Subg. *Rufescentia* Niskanen & Liimat. (99% ML bootstrap, 1.00 BPP): including *H. ellipsosporum*, *H. umbilicatum*, *H. cuspidatum*, *H. quebecense*, *H. oregonense*, *H. subconnatum*, *H. atlanticum*, *H. longibasidium*, *H. itachiharitake*, *H. paucispinum*, *H. mulsicolor*, *H. submulsicolor*, *H. canadense*, *H. longipes*, *H. melitosarx*, *H. berkeleyanum*, *H. subberkeleyanum*, *H. ventricosum*, *H. rufescens*, *H. subrufescens*, *H. aerostatisporum*, *H. ovoideisporum*, *H. subovoideisporum*, *H. jussii*, *H. pallidocroceum*, *H. melleopallidum*, *H. albertense*, *H. ferruginescens*, *H. magnorufescens*, *H. tangerinum*, *H. minospororufescens*, *H. crocidens*, *H. mcnabbianum*, *H. crassipedum*, *H. fulvostriatum*, *H. roseotangerinum*, *H. fibrillosum*.

Subg. *Hydnum* L. (95% ML bootstrap, 0.99 BPP): including *H. repando-orientale*, *H. subalpinum*, *H. boreorepandum*, *H. repandum*, *H. neorepandum*, *H. washingtonianum*, *H. sphaericum*, *H. sinorepandum*, *H. vagabundum*, *H. olympicum*, *H. slovenicum*, *H. subolympicum*, *H. flosculoides* sp. nov.

Clade *Insulana* (100% ML bootstrap, 1.00 BPP): including 18 *Hydnum* sp. collected from Australasia.

Subg. *Pallida* Niskanen & Liimat. (90% ML bootstrap, 0.99 BPP) including *H. tottoriense*, *H. flabellatum*, *H. pallidomarginatum*, *H. khanspurense*, *H. ibericum*, *H. flavosquamosum*, *H. erectum*, *H. albopallidum*, *H. subtilior*, *H. vesterholtii*.

Subg. *Brevispina* T. Cao & H.S. Yuan (98% ML bootstrap): including *H. tenuistipitum*, *H. alboluteum*, *H. microcarpum*, *H. alboaurantiacum*, *H. brevispinum*, *H. minum*.

Subg. *Alba* Niskanen & Liimat. (89% ML bootstrap, 0.90 BPP): including *H. robustum* sp. nov., *H. pinicola*, *H. cremeoalbum*, *H. subcremeoalbum*, *H. albomagnum*, *H. reginae*.

Subg. *Alba* s.l. Niskanen & Liimat. (99% ML bootstrap, 1.00 BPP): including *H. bifurcatum*, *H. tomaense*, *H. treui*, *H. roseoalbum*, *H. zongolicense*.

Incertae sedis: including *H. aurantiascens*, *H. flavidocanum*, *H. albidum*, *H. orientalbidum*., *H. elatum*.

### Taxonomy

**Figure 2.**
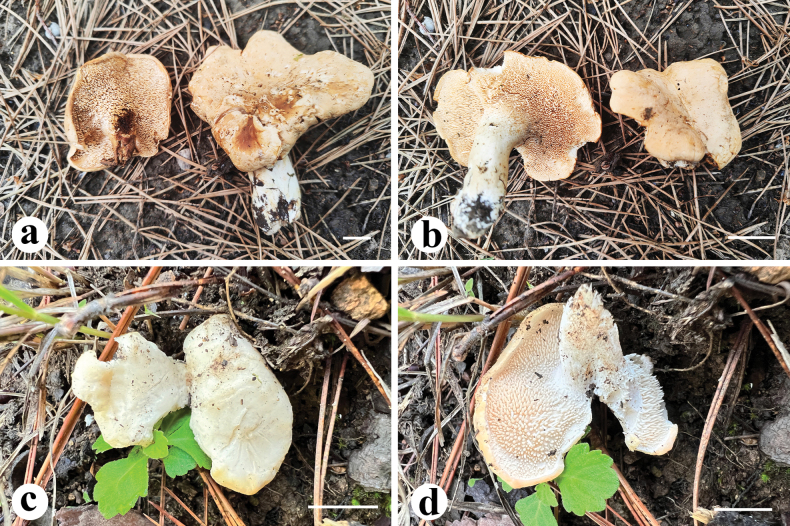
Fresh basidiomata of two new *Hydnum* species. **a, b***H. flosculoides* (Cui 23128, holotype) **c, d***H. robustum* (Cui 22893, holotype). Scale bars: 2 cm (**a, b**); 1 cm (**c, d**).

**Figure 3.**
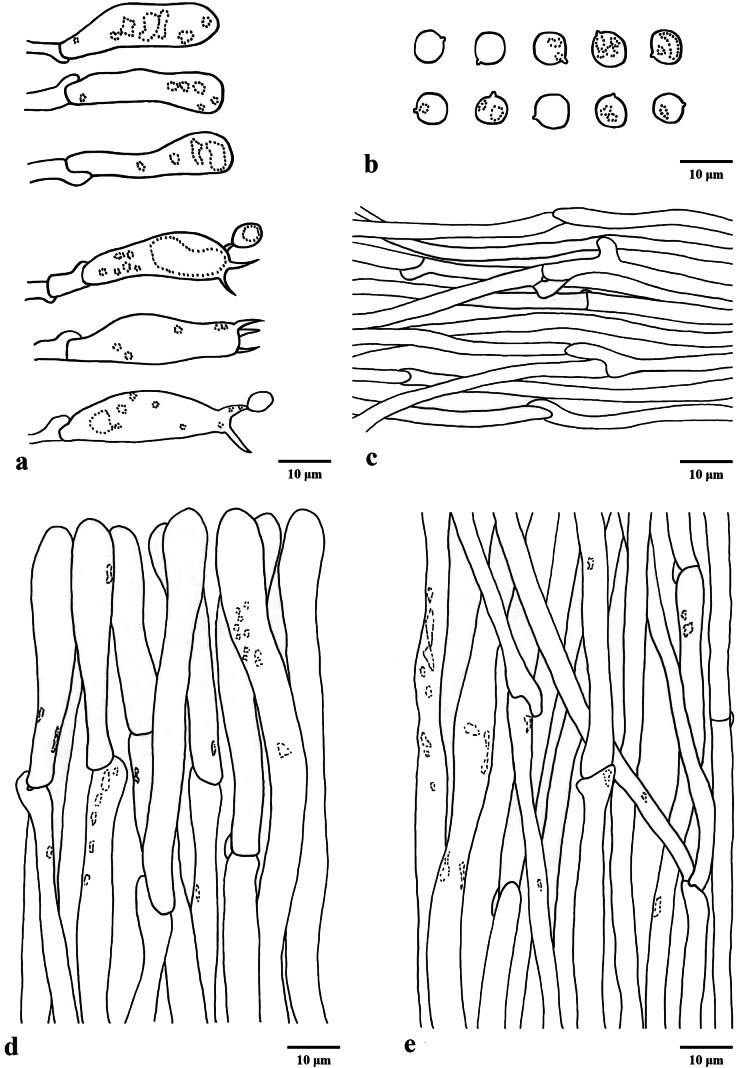
Microscopic features of *Hydnum flosculoides* (Cui 23128, holotype). **a** Basidia and basidioles **b** Basidiospores **c** Hyphae of spines **d** Pileipellis terminal hyphae with clamp connections **e** Stipitipellis hyphae with clamp connections. Scale bars: 10 µm (**a–e**).

**Figure 4.**
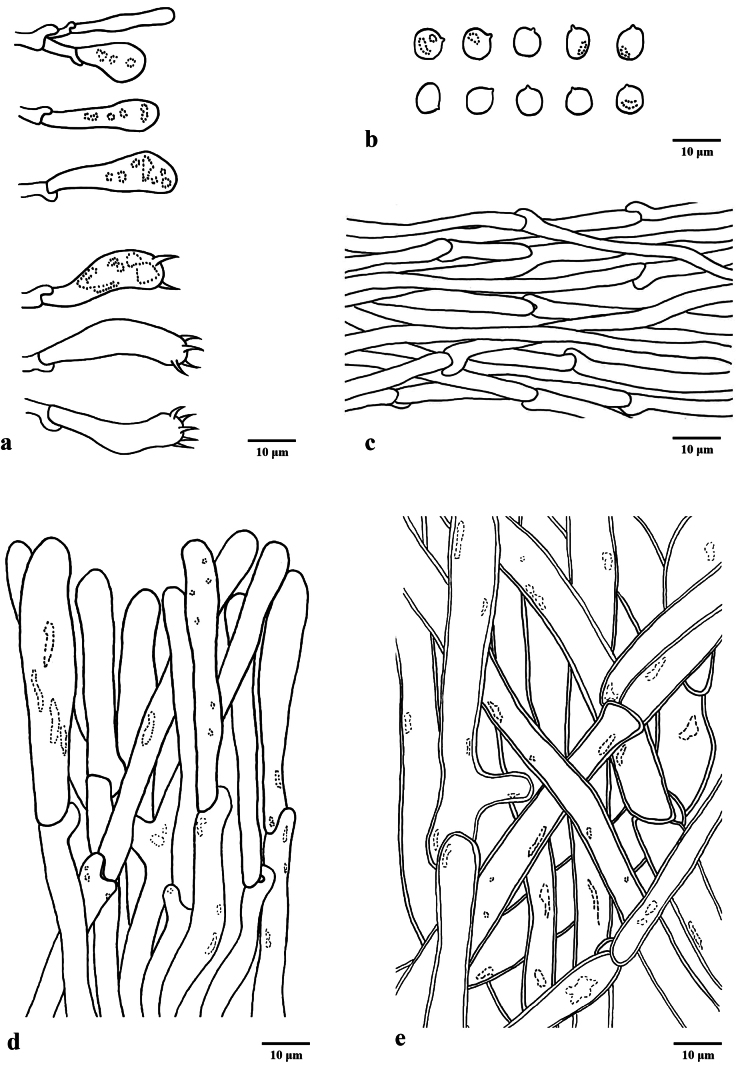
Microscopic features of *Hydnum robustum* (Cui 22893, holotype). **a** Basidia and basidioles **b** Basidiospores **c** Hyphae of spines **d** Pileipellis terminal hyphae with clamp connections **e** Stipitipellis hyphae with clamp connections. Scale bars: 10 µm (**a–e**).

One new clade and two new species of *Hydnum* are presented according to the morphological and phylogenetic evidence. Here, the fresh basidiomata of two new species are displayed (Fig. 2), and the description and illustrations of them are also provided (Figs 3, 4).

#### Clade Insulana

Taxon classificationAnimaliaCantharellalesHydnaceae

3EAF0EAE-E3C7-517D-8BDD-8E77DCA6C517

##### Etymology.

*Insulana* (Lat.), refers to the insular geographic provenance of taxa in this subgenus.

##### Notes.

The specimens in this clade were mostly collected from insular ecosystems of Oceania and Malay Archipelago, such as Australia, New Caledonia, New Zealand, Malaysia and Singapore. According to their distinct geographical distribution and phylogenetic relationship (96% ML bootstrap, 1.00 BPP) (Fig. 1), clade *Insulana* was presented to accommodate these specimens, which is consistent with previous findings in Feng et al. 2016, Niskanen et al. 2018 and Sugawara et al. 2022. Due to the lack of researchable specimens, it is currently proposed as a new clade rather than a new subgenus.

#### Hydnum flosculoides

Taxon classificationAnimaliaCantharellalesHydnaceae

Y.H. Xu, Y.F. Sun & B.K. Cui sp. nov.

884FF118-8723-51E3-B45B-01F11F6E6158

856506

Figs 2 a, b, 3

##### Etymology.

*Flosculus* (Lat.), refers to the flower-like appearance of the species with undulate pileus.

##### Type.

CHINA. Yunnan Province, Mangshi, alt. ca. 1200 m; 31 Aug. 2023, Yunnan Shanshifu leg., on ground of forest mixed by bamboo, *Quercus* and *Pinus*, Cui 23128 (BJFC, holotype).

##### Diagnosis.

Characterized by its flower-like basidiomata with large and undulate pileus (up to 110 mm wide), orange hymenophore with decurrent spines.

##### Description.

Basidiomata solitary to gregarious, fleshy and moderately firm when fresh, becoming leathery upon drying. Pilei up to 110 mm wide, round to reniform, plano-convex when young; ellipsoid or irregularly round, umbilicate at center or infundibuliform when old. Pileus surface dry, subglabrous, azonate, pale orange to orange (5A3–6B8) when fresh, yellow-orange to grayish orange (4B8–5B4) when dry. Pileus margin thin, entire, undulate, strongly decurved, same color or sometimes darker than the pileus surface. Context 1–5 mm thick, white to cream white (4A2). Hymenophore hydnoid, spines decurrent, crowded, evenly distributed; surface orange-white (5A2) when fresh, light orange or brown (5A4/6D4) when dry; subulate, acute, straight to somewhat flexuous, solitary, 1–3.5 mm long, shortest near the pileus margin, 3–4 per mm, brittle when dry. Stipes central, 40–70 mm long, 16 mm wide, subcylindrical, solid; surface glabrous, white to cream white (4A2); stipe base enlarged and covered with a little amount of white basal mycelium. Odor mild and fruity.

Basidiospores subglobose to broadly ellipsoid, hyaline, thin-walled, smooth, some with granular contents, IKI–, (5.4–)5.6–6.7(–6.9) × (4.7–)5–5.8(–6.4) μm, av. 6.14 ± 0.31 × 5.37 ± 0.30 μm, Q = 1.05–1.23 (n = 90/3), Qm = 1.14 ± 0.06. Under SEM, the basidiospores smooth without any ornamentation, each bearing a distinct hilar appendix at the attachment point. Basidia subcylindrical to clavate, 27–39 × 6.5–8.5 μm, sometimes containing granular contents; sterigmata 2–4, up to 4–8 μm long, 1–1.2 μm wide at base, slightly curving. Basidioles numerous, subcylindrical to subclavate, 25–36 × 6.5–7 μm. Cystidia absent. Hyphae of spines 2.3–3.7 µm wide, cylindrical, thin-walled, smooth, hyaline, subparallel, enlarged at hyphal apex or septum, up to 4.7–9.9 μm, greenish yellow in KOH. Pileipellis composed of loosely interwoven erect to cylindrical hyphae, occasionally containing granular contents, 4–7.5 µm wide, enlarged at hyphal apex to 6–11 μm. Stipitipellis composed of subcylindrical hyphae, thin-walled, interwoven, 3–3.6 μm wide, terminal elements rounded at apex, sometimes containing hyaline oily droplets. Clamp connections present in all tissues.

##### Additional materials examined.

CHINA. Yunnan Province, Chuxiong, Zixi Mount., alt. ca. 1500 m, 11 Aug. 2023, Bao-Kai Cui leg., on ground of forest mixed by *Fagus* and *Pinus*, Cui 22857 (BJFC). Yunnan Province, Mangshi, alt. ca. 1200 m, 31 Aug. 2023, Yunnan Shanshifu leg., on ground of forest mixed by bamboo, *Quercus* and *Pinus*, Cui 23129 (BJFC).

##### Distribution.

Known only from China.

##### Notes.

In the phylogenetic tree, *Hydnum flosculoides* was involved in subg. *Hydnum* forming a separate group (98% ML bootstrap, 1.00 BPP; Fig. 1). It is closely related to *H. sphaericum* which both have a cream to orange pileus surface, crowded spines, thin-walled hyphae with clamps in the spines. However, *H. sphaericum* differed from *H. flosculoides* in having a subglobose and smaller pileus (25–35 mm), smaller stipes (18–25 × 5–8 mm, non-decurrent to subdecurrent spines in white, white (3A1–4A1) hymenophore when fresh and bigger basidiospores (8.0–8.8 × 6.5–7.5 μm; Cao et al. 2021).

#### Hydnum robustum

Taxon classificationAnimaliaCantharellalesHydnaceae

Y.H. Xu, Y.F. Sun & B.K. Cui sp. nov.

8F11BFFA-37A7-53AC-8800-5728FC53BB54

856511

Figs 2 c, d, 4

##### Etymology.

*Robustum* (Lat.), refers to the robust basidiomata.

##### Diagnosis.

Characterized by its robust basidiomata and narrow hyphae on pileipellis (2.5–4.5 µm wide).

##### Type.

CHINA. Yunnan Province, Lanping County, Luogujing Nature Reserve, alt. ca. 1900 m, 13 Aug. 2023, Yi-Fei Sun leg., on ground of *Pinus* forest, Cui 22893 (BJFC, holotype).

##### Description.

Basidiomata gregarious, robust, 16–30 mm high, fleshy when fresh, becoming ceraceous and cocky upon drying. Pilei up to 35 mm wide, irregularly round or semicircular, convex to plano-convex when young, infundibuliform when old, shallowly depressed in the center; surface glabrous, azonate, smooth to irregularly bumpy; white to cream (4A2), tinged pale yellow (4A2–4A4) when fresh; turning to orange-brown to reddish brown (7E7–8E7) when dry. Pileus margin thick, entire, strongly decurved, flatten, concolorous with pileus surface. Context 1–2 mm thick, white to cream white (4A2). Hymenophore hydnoid, spines subdecurrent, crowded, 3–4 spines/mm^2^ when fresh, evenly distributed, subulate, acute, solitary, up to 2.5 mm long and up to 0.3 mm diam; surface white to yellowish white (4A2) when fresh, light brown (6D7/6D8) when dry. Stipes central, robust, up to 25 mm long, 5–15 mm wide, subcylindrical, solid, glabrous, concolorous with spines surface; unchanging when handled; stipe base enlarged and covered with a small amount of white basal mycelium. Odor mild and fruity.

Basidiospores subglobose to broadly ellipsoid or ovoid, hyaline, thin-walled, smooth, some with granular contents, IKI–, (3.8–)4.1–5.2(–5.8) × (3–)3.2–4.6(–4.9) μm, av. 4.62 ± 0.33 × 3.94 ± 0.38, Q = 1.01–1.37 (n = 60/2), Qm = 1.18 ± 0.09. Under SEM, the basidiospores smooth without any ornamentation, each bearing a distinct hilar appendix at the attachment point. Basidia subcylindrical or subclavate to clavate, (20–)22–30(–33) × (3–)4–6.5 μm, usually contains hyaline oily droplets; sterigmata 2–5, 2–5 μm long, 1–1.5 μm wide at base, slightly curving. Basidioles numerous, subcylindrical to subclavate, enlarge at apex, smaller or the same size with basidia, frequently covered with granular substance at the apex. Cystidia absent. Hyphae of spines 2.5–4.5 μm, filamentous, hyaline to pale yellow, thin-walled, apex cylindrical, interwoven, frequently branched, pale yellow in KOH. Pileipellis a mixocutis, hyaline to pale yellow, composed of hyphae 2.5–4.5 µm wide, thin to slightly thick-walled, cylindrical to irregular, slightly inflated at apex. Stipitipellis composed of subcylindrical hyphae, slightly thick-walled, interwoven, 1.5–3 μm wide, yellowish brown, sometimes containing yellowish cytoplasmic pigment. Clamp connections present.

##### Additional materials examined.

CHINA. Yunnan Province, Lanping County, Luogujing, alt. ca. 1900 m, 13 Aug. 2023, Yi-Fei Sun leg., on ground of *Pinus* forest, Cui 22901 (BJFC). Yunnan Province, Lanping County, Luogujing, 13 Aug. 2023, Yi-Fei Sun leg., on ground of *Pinus* forest, Cui 22904 (BJFC). Yunnan Province, Lanping County, Luogujing, alt. ca. 1900 m, 13 Aug. 2023, Yi-Fei Sun leg., on ground of *Pinus* forest, Cui 22905 (BJFC). Yunnan Province, Lijiang City, Yulong County, Jiuhe, alt. ca. 2400 m, 1 September 2020, Ming Zhang leg., on ground of mixed forests mainly dominated by *Fagaceae* and *Pinus* trees, GDGM83047. Hubei Province, Huanggang City, Luotian, alt. ca. 800 m, 22 October 2023, Xiaomei Peng leg., on ground of mixed forests mainly dominated by *Fagaceae* and *Pinus* trees, GDGM93020.

##### Distribution.

Known only from China.

##### Notes.

The specimens numbered as GDGM83047, GDGM93020 and HKAS57714 collected from southern subtropical China were firstly recognized as *Hydnum pinicola* R. Sugaw. & N. Endo, which is originally described from Japan, having whitish to cream basidiomata, pale yellow or pale orange spines, and relatively smaller basidiospores (4.5–5.5 × 4–5 µm; Sugawara et al. 2022). However, the addition of our new specimens revealed that the material from China formed a separate lineage with strong support in the multi-gene phylogenetic tree (87% in ML bootstrap, 0.99 BPP). Further morphological study revealed that *H. robustum* has a wider stipe (up to 25 mm) combined with a lower basidioma (16–30 mm) and shorter spines (up to 3 mm), resulting in a robust appearance. Moreover, the pileipellis and stipitipellis of *H. robustum* show a broader range of hyphal diameters (pileipellis 2–17 µm, stipitipellis 2–12 µm).

### Meta-analysis of ITS sequences

**Figure 5.**
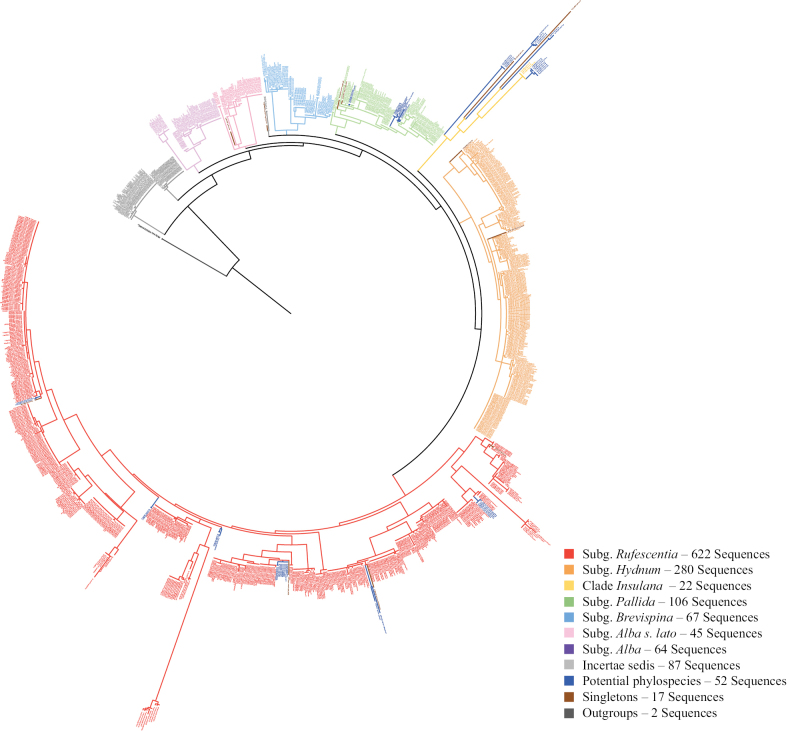
Maximum likelihood tree of *Hydnum* based on ITS sequences.

The phylogeny of 1,293 ITS sequences reveals seven monophyletic clades, including Subg. *Rufescentia*, Subg. *Hydnum*, Subg. *Pallida*, Subg. *Brevispina*, Subg. *Alba*, Subg. *Alba* s.l. and Clade *Insulana*, and four clades labeled as Incertae sedis, which shows a topology and subgenera composition similar to that obtained from the multi-gene phylogenetic analysis (Fig. 5, Suppl. material 1: table S3). The phylogenetic tree alignment and topology is available in Suppl. materiasl 9–11.

### Subg. *Rufescentia* Niskanen & Liimat.

Subg. *Rufescentia* corresponded to a well-supported clade (99% ML bootstrap), including 43 phylogenetic groups: 46 identified species (593 sequences), seven well-supported phylospecies (24 sequences), and five single-sequence singletons. In addition, 34 sequences were derived from environmental samples. The taxa in this subgenus were mostly derived from temperate or tropical regions of the Northern Hemisphere (e.g., China, India, Vietnam, etc.) and Oceania (Australia and New Zealand; Suppl. material 1: table S3).

### Clade *Insulana*

A total of 22 sequences were involved in this clade and formed a well-supported clade in the phylogenetic tree (97% ML bootstrap). It was formed mostly by taxa from Australia, New Zealand and Malaysia. Beyond it, the subgenus appeared to harbor significant undescribed diversity, collectively represented by 15 sequences designated as *Hydnum* sp. *Insulana*. This was not a single entity but rather a collection of at least seven distinct phylogenetic lineages distributed across Australia, New Zealand, Singapore, Malaysia, and New Caledonia. The existence of these multiple, well-supported but unnamed clades indicated that the true species diversity within clade *Insulana* is substantially underestimated and awaits formal taxonomic study (Suppl. material 1: table S3).

### Subg. *Hydnum* L.

Subg. *Hydnum* was strongly supported (95% ML bootstrap) and included 14 phylogenetic groups: 14 identified species with 280 sequences and three singletons. In total, three sequences were derived from environmental samples. The species grouped in this subgenus had a worldwide distribution (Suppl. material 1: table S3).

The core of the subgenus was the *H. repandum* species complex, represented by 56 sequences primarily from a wide range of European localities. This group was found in diverse habitats, from oak woodlands to *Picea* and *Pinus* plantations, typically on rich or calcareous soils. The lack of a high support value for this clade in the analysis suggested that *H. repandum* s.l. is not a single monophyletic species but rather a collection of closely related lineages. This taxonomic confusion was evident throughout the subgenus, as numerous distinct clades contained sequences originally mislabeled as *H. repandum*.

### Subg. *Pallida* Niskanen & Liimat.

Subg. *Pallida* (100% ML bootstrap) included a total of 106 sequences corresponding to 11 identified species with 91 sequences, three well-supported phylospecies with 11 sequences, and four singletons. Of these, one sequence was derived from an environmental sample. The taxa in this subgenus were mostly derived from Eurasia and North America (Suppl. material 1: table S3).

Significant undescribed diversity exists in this subgenus, represented by a collection of 15 sequences labeled *Hydnum* sp. *Pallida*. These sequences are distributed among at least seven distinct lineages from China and Japan, several of which were strongly supported (e.g., *Hydnum* sp. *Pallida* 3 with 98% ML bootstrap support and *Hydnum* sp. *Pallida* 4 with 85% ML bootstrap support) and were clear candidates for new species. This indicated that the species inventory for Subgenus *Pallida*, particularly in East Asia, was still far from complete.

### Subg. *Brevispina* T. Cao & H.S. Yuan

Subg. *Brevispina* was well supported (94% ML bootstrap), and included 67 sequences corresponding five identified species. It was majorly formed by specimens collected from China and Japan except *H. alboaurantiacum*, which was only found in North America (USA, Canada) to Central America (Belize, Mexico) (Suppl. material 1: table S3). It inhabited diverse environments, including mixed woods, deciduous woodlots, and cloud forests associated with *Quercus*, *Pinus*, and *Fagus*. The most prominent of these was the unresolved *H. tenuistipitum* - *H. brevispinum* phylogenetic group, which was represented by 28 sequences in a moderately supported clade (78% ML bootstrap). All taxa in this group originated from China, were found in mixed forests with *Pinus armandi* and *Quercus*, as well as in other angiosperm forests. The ITS data were insufficient to separate *H. tenuistipitum* and *H. brevispinum*, and the clade was a composite of sequences submitted under these two names, as well as many labeled simply as *Hydnum* sp. or derived from an environmental sample.

### Subg. *Alba* Niskanen & Liimat.

Subg. *Alba* was moderately supported (87% ML bootstrap), and predominantly composed of taxa from the temperate regions of the Northern Hemisphere and Oceania (Papua New Guinea). It included 64 sequences comprising 5 identified species (Suppl. material 1: table S3).

The East Asian species in this group include *H. cremeoalbum* (28 sequences, 97% ML bootstrap), found in mixed forests with *Quercus* in Japan, China, and Korea; *H. pinicola* (8 sequences, 78% ML bootstrap), associated with *Pinus* and *Quercus* in Japan and Korea; and *H. robustum* (9 sequences, 77% ML bootstrap), from *Pinus* forests in China. The primary European representative was *H. reginae*, a highly supported species (13 sequences, 100% ML bootstrap) from *Fagus* and mixed forests on calcareous soils. The Americas were represented by a single species in this group, *H. albomagnum* (6 sequences, 86% ML bootstrap), found in broad-leaved and mixed pine-oak forests in the USA and the Dominican Republic.

### Subg. *Alba* s.l. Niskanen & Liimat.

Subg. *Alba* s.l. (75% ML bootstrap), appeared to represent a rapid radiation of species, primarily in East Asia and Mexico. It included 45 sequences comprising 7 identified species, one well-supported phylospecies with four sequences, and two singletons (Suppl. material 1: table S3). The most sampled was *H. minum* (21 sequences, 100% ML bootstrap), from angiosperm and mixed forests in Japan, China, and Korea. China, in particular, hosted a remarkable diversity of these species, including *H. treui* (3 sequences) from subtropical moist lowland forests, *H. roseoalbum* (2 sequences, 96% ML bootstrap) from broad-leaved forests, *H. bifurcatum* (4 sequences, 92% ML bootstrap) from *Quercus mongolica* forests, and *H. flavidocanum* (3 sequences, 99% ML bootstrap) from mixed angiosperm and *Pinus* forests.

### Incertae sedis

This phylogenetic group represented an assemblage of species, included three identified species (83 sequences), and one singleton (Suppl. material 1: table S3).

The neotropical species *H. aurantiascens* (2 sequences, 99% ML bootstrap), was found in *Quercus* and pine-oak forests in Belize. *Hydnum albidum*, a highly supported species (33 sequences, 97% ML bootstrap) with a broad distribution from North America (USA, Canada) through Central America (Belize, Costa Rica, Mexico) down to South America (Colombia). It was found in a variety of habitats, including mixed woods and montane cloud forests. Distinct from its American counterpart, *H. orientalbidum* was a highly supported East Asian species (51 sequences, 98% ML bootstrap). This species was common in coniferous, mixed, and broadleaved forests in Japan, China, and the Republic of Korea. Finally, a single environmental sequence from Panama, was labeled *Hydnum* sp. Incertae sedis 1.

### Global habitat and species summary based on analyzed ITS sequences data

Based on the ITS meta-analysis, the global geographic distribution and habitat preferences for all *Hydnum* species were systematically summarized (Table 2). East Asia (particularly China) and North America were the two principal centers of species diversity, whereas Australasia and the Neotropics exhibited a higher proportion of continent-restricted species.

**Table 2.** Global habitat and species summary for *Hydnum* based on analyzed ITS data.

Region (Countries)	Habitat Summary	Recorded Taxa (ITS)
North America	Primarily temperate and boreal habitats, including damp coniferous, mixed, and hardwood forests on various soil types, as well as old-growth forests and mossy substrates.	*H. albidum*, *H. alboaurantiacum*, *H. albertense*, *H. atlanticum*, *H. aerostatisporum*, *H. canadense*, *H. cuspidatum*, *H. cf. umbilicatum*, *H. ferruginescens*, *H. ibericum*, *H. magnorufescens*, *H. melleopallidum*, *H. melitosarx*, *H. mulsicolor*, *H. neorepandum*, *H. olympicum*, *H. oregonense*, *H. quebecense*, *H. repandum*, *H. subconnatum*, *H. subolympicum*, *H. submulsicolor*, *H. subtilior*, *H. umbilicatum*, *H. vagabundum*, *H. washingtonianum*.
(USA & Canada)		
Central America & Caribbean	Neotropical habitats including montane cloud forests, subtropical moist mixed forests, and pine-oak forests.	*H. albidum*, *H. alboaurantiacum*, *H. aurantiascens*, *H. cuspidatum*, *H. fibrillosum*, *H. formosum*, *H. mulsicolor*, *H. subtilior*, *H. vagabundum*, *H. zongolicense*.
(Mexico, Belize, Costa Rica, Panama, Dominican Republic)		
South America	Montane cloud forests and mixed woods.	*H. albidum*, *H. repandum* (environmental sample).
(Colombia, Venezuela)		
Europe	Wide range of temperate habitats including coniferous (*Picea*, *Pinus*), broad-leaved (*Fagus*, *Quercus*), and mixed forests, often on rich or calcareous soils.	*H. umbilicatum*, *H. ellipsosporum*, *H. rufescens*, *H. melitosarx*, *H. mulsicolor*, *H. persicinum*, *H. ovoideisporum*, *H. subovoideisporum*, *H. magnorufescens*, *H. jussii*, *H. boreorepandum*, *H. repandum*, *H. slovenicum*, *H. ibericum*, *H. vesterholtii*, *H. reginae*.
(Andorra, Austria, Bulgaria, Denmark, Estonia, Finland, France, Germany, Italy, Montenegro, Norway, Poland, Portugal, Slovenia, Spain, Sweden, Switzerland, UK)		
Russia	Coniferous or mixed forests.	*H. umbilicatum*, *H. jussii*, *H. repandum*.
Africa	Found in broad-leaved and coniferous forests on calcareous soil in Tunisia.	*H. ovoideisporum*, *H. magnorufescens*, *H. repandum*.
(Tunisia)		
Asia	Extremely diverse habitats from temperate to subalpine forests, including broad-leaved, coniferous, and mixed forests dominated by *Fagaceae*, *Pinaceae*, and *Betulaceae*.	*H. albidum*, *H. albomagnum*, *H. albomarginatum*, *H. alboluteum*, *H. albopallidum*, *H. berkeleyanum*, *H. bifurcatum*, *H. boreorepandum*, *H. brevispinum*, *H. crassipedum*, *H. cremeoalbum*, *H. cremeum*, *H. ellipsosporum*, *H. erectum*, *H. flabellatum*, *H. flavidocanum*, *H. flavosquamosum*, *H. flosculoides*, *H. fulvostriatum*, *H. ibericum*, *H. itachiharitake*, *H. jussii*, *H. longibasidium*, *H. longipes*, *H. magnorufescens*, *H. melitosarx*, *H. microcarpum*, *H. minum*, *H. minospororufescens*, *H. orientalbidum*, *H. pallidocroceum*, *H. pallidomarginatum*, *H. paucispinum*, *H. pinicola*, *H. repandum*, *H. repando-orientale*, *H. robustum*, *H. roseoalbum*, *H. roseotangerinum*, *H. rufescens*, *H. sinorepandum*, *H. sphaericum*, *H. subalpinum*, *H. subberkeleyanum*, *H. tangerinum*, *H. tenuistipitum*, *H. tomaense*, *H. tottoriense*, *H. treui*, *H. umbilicatum*, *H. ventricosum*.
(East: China, Japan, Republic of Korea)		
Asia	Found in mixed broad-leaved and coniferous forests.	*H. berkeleyanum*, *H. khanspurense*, *H. rufescens*.
(South: India, Pakistan)		
Australasia	Found in subtropical and tropical moist lowland forests, on the ground under native trees and shrubs such as *Eucalyptus* and *Nothofagus*.	*H. elatum*, *H. treui*, *H. crocidens*, *H. mcnabbianum*.
(Singapore, Malaysia, Australia, New Zealand, New Caledonia)		

In terms of habitat, the genus *Hydnum* demonstrated broad ecological range, with species inhabiting ecosystems ranging from boreal coniferous and temperate mixed forests to tropical montane cloud forests, across various soil types from acidic to calcareous.

## Discussion

The taxonomy of *Hydnum* has become increasingly complex with expanding geographic sampling; however, previous studies have mainly focused on taxa from the Northern Hemisphere, leaving most taxa from the Southern Hemisphere with unresolved taxonomic ambiguity. Since Feng et al. (2016) discovered three phylogenetic species from Australia and New Zealand (*Hydnum* sp. 17–19 BF-2016), and established the first phylogenetic study based on ITS sequences, subsequent studies have systematically excluded this clade due to its strong ITS divergence from continental relatives (Niskanen et al. 2018; Sugawara et al. 2022). With the inclusion of our new specimen Cui 16706, a well-supported lineage was formed with the previously published specimens *Hydnum* sp. GD1588, PERTH08072957, PERTH07830742 and BH2066F (Fig. 1). However, due to the lack of available materials, we are unable to conduct sufficient analyses to formally propose new species or subgenus. To emphasize its distinctive insular distribution across Oceania and the Malay Archipelago, we here designate this assemblage as clade *Insulana*. This insular pattern contrasts with the predominantly holarctic and continental distributions typical of many other *Hydnum* species. In addition, based on comprehensively morphological and phylogenetic studies, two additional new *Hydnum* species were presented, *H. flosculoides* and *H. robustum*. To date, there are 84 confirmed species of *Hydnum* in the world, of which 33 species have been recorded in China.

Besides the multi-gene phylogenetic construction, a metadata phylogenetic analysis of 1,293 ITS sequences is also conducted in this study. Both analyses divided the genus *Hydnum* into seven monophyletic clades (Figs 1, 5). The results were similar to the previous studies (Niskanen et al. 2018; Swenie et al. 2018; Sugawara et al. 2022; Justo et al. 2023; Qin et al. 2024; Zhang et al. 2024). As for subg. *Alba s.lato*, it is used to denote pale *Hydnum* species that are distributed close to subg. *Alba* and originally included *H. treui*, *H. minum* and *H. zongolicense* (Niskanen et al. 2018). In this study, *H. treui*, *H. minum* and *H. zongolicense* together form a well-supported clade. In agreement with recent studies, the morphologically similar taxa *H. albidum* and *H. orientalbidum* are consistently resolved as a separate, stable clade. Therefore, following Niskanen et al. (2018), we provisionally circumscribe subg. *Alba s. lato* to include *H. treui*, *H. minum*, *H. zongolicense*, and related taxa, while treating *H. albidum* and *H. orientalbidum* as incertae sedis.

While both multi-gene and ITS analyses provided a robust subgeneric framework, they exhibit notable discordance at the species level, highlighting the dual role of the ITS barcode. On one hand, its limitations are clear: a total of 21 species that were distinctly separated in the multi-gene tree could not be distinguished by ITS alone. A major discrepancy was the placement of *H. minum*, which our multi-gene analysis strongly supports within subg. *Brevispina* (96% ML bootstrap, 0.86 BPP; Fig. 1), consistent with Qin et al. (2024), whereas ITS phylogenies place *H. minum* in subg. *Alba* s.l., distinct from other *Brevispina* members (*H. tenuistipitum*, *H. alboluteum*, etc.; Fig. 5, Suppl. material 1: table S3). This conflict confirms that multi-gene phylogenetic analyses exhibit higher effectiveness in identifying species of *Hydnum*, other gene fragments still need to significantly improve its success rate of amplification, particularly *tef1α*, *rpb2*, mtSSU and so on.

On the other hand, ITS metadata analysis provides a comprehensive overview of the genus, enabling the correction of historical taxonomic errors based only on morphology and revealing substantial hidden diversity. Biogeographically, our ITS metadata analysis provides a comprehensive molecular confirmation that *Hydnum* comprises ectomycorrhizal fungi with a widespread global distribution, documented across six continents, predominantly in subtropical and temperate regions of Asia, North America and Europe. More importantly, the data uncover a finer and stricter pattern of intercontinental endemism than previously understood. For example, *H. albidum* is genetically distinct and restricted to the Americas, differing from its morphologically similar East Asian counterpart, *H. orientalbidum*. An analogous pattern of taxonomic revision applies to *H. vesterholtii* and *H. albomagnum*. Chinese specimens previously misidentified as the European *H. vesterholtii* are reassigned to several East Asian lgroups: *H. pallidomarginatum*, *H. ibericum* and *H. erectum*, consistent with Su et al. (2024). Similarly, specimens in Chinese misassigned to the North American species *H. albomagnum* are clearly reallocated to the *H. cremeoalbum* group.

In contrast, ITS metadata analysis shows that only a few *Hydnum* species exhibit truly widespread, intercontinental distributions. The most prominent example is *H. umbilicatum*, a classic Holarctic species with extensive records across North America, Europe, and Asia. Similarly, *H. melitosarx* and *H. jussii* also exhibit clear Holarctic distribution patterns. The distribution of *H. vagabundum* is particularly noteworthy, as its range extends from North America and China into the Neotropics of Central America, highlighting its remarkable ecological adaptability. While primarily European, species like *H. repandum* and *H. magnorufescens* show a broader geographic potential with records extending into Asia and North Africa (Suppl. material 1: table S3).

Our comprehensive dataset also helps to clarify specific taxonomic debates. For example, Su et al. (2024) proposed that *H. flabellatum* is a synonym of *H. pallidomarginatum*, citing overlapping morphology and what they determined to be poor-quality ITS sequence data for the *H. flabellatum* type. However, from both the ITS metadata and multi-gene analyses, these two taxa still show obvious molecular differences whether in branch length (Figs 1, 5) or base differences (Suppl. materials 2, 9). This molecular separation is supported by clear morphological differences noted by Cao et al. (2021), including pileus texture, margin color, stipe position, and spine density. Therefore, we maintain that *H. flabellatum* and *H. pallidomarginatum* are distinct species before more specimens are studied. Moreover, in a related finding, our analysis revealed that the ITS sequence for type specimen *H. flavosquamosum* (PQ287672, voucher MHKMU LP Tang 3454) clusters with a sequence identified by Su et al. (2024) as *H. pallidomarginatum* (PQ287662, voucher MHKMU LP Tang 3453) with high support in both the ITS (86% ML bootstrap; Fig. 5) and multi-gene trees (96% ML bootstrap; Fig. 1). We provisionally treat them as the same species here but recommend further morphological examination of the voucher specimens to confirm this conclusion.

From an ecological niche perspective, *Hydnum* species, as obligate ectomycorrhizal fungi, occupy a broad range of forest niches, but their distribution shows a strong association with specific host plant families, particularly *Pinaceae* and *Fagaceae*. Globally, their typical habitats are the temperate and boreal coniferous, broad-leaved, and mixed forests of the Northern Hemisphere, as well as the montane cloud forests of the tropics and subtropics. The data show that symbiotic relationships with dominant tree genera such as *Pinus*, *Picea*, *Abies*, *Tsuga*, *Quercus*, and *Fagus* are the most commonly documented hosts for *Hydnum*. These species typically grow on humus-rich, moss-covered soil or in leaf litter, while some European species (like *H. ovoideisporum* and *H. vesterholtii*) show a clear preference for calcareous soils. Overall, the niche preferences of the genus *Hydnum* are a key factor in explaining its global biogeographic patterns (Suppl. material 1: table S3).

Finally, the dataset reveals clear research biases, with extensive sampling in North America, Europe, and East Asia, but significant gaps in regions like Africa, most of South America, and Southeast Asia. The sparse but notable records still exist from Africa (e.g., *H. ovoideisporum*, *H. magnorufescens*, *H. repandum* from Tunisia) and South America (e.g., *H. albidum* and an environmental sequence of *H. repandum* from Colombia and Venezuela), as well as from South Asia (e.g., *H. berkeleyanum*, *H. khanspurense*, *H. rufescens* from India and Pakistan) (Table 2). These occurrences demonstrate that *Hydnum* has a wide distribution range, but current data remain insufficient to evaluate true species diversity or biogeographic patterns. Consequently, targeted field surveys, voucher-based sequencing, and region-specific environmental sampling will be essential for building a more complete understanding of global *Hydnum* diversity.

## Conclusions

In summary, two new species of *Hydnum* were proposed in this study with detailed morphology illustrations and descriptions. And the multi-gene and ITS phylogenetic analyses were conducted separately, which clarified the phylogenetic relationships among clades and species in *Hydnum*, highlighted the respective advantages of each approach, and corrected the issues with ITS sequences in public databases. Based on these meaningful findings, the taxonomic framework of *Hydnum* has been effectively improved, and its species diversity has been enriched.

Future research on *Hydnum* should prioritize resolving the ambiguous groups, especially Clade *Insulana*, unrecognized clades: Incertae sedis 1, 2, 3 and the unrecognized taxa like *Hydnum* sp. Expanding geographic sampling and supplementing genetic data are essential to better understand *Hydnum* diversity and perfect its taxonomic system.

## Supplementary Material

XML Treatment for Insulana

XML Treatment for Hydnum flosculoides

XML Treatment for Hydnum robustum

## Additional information

### Use of AI

No use of AI was reported.

### Adherence to national and international regulations

All the fungal strains used in this study have been legally obtained, respecting the Convention on Biological Diversity (Rio Convention).

### Author ORCIDs

Yi-Hua Xu https://orcid.org/0009-0007-8997-2517

Long Zeng https://orcid.org/0009-0004-0124-459X

Tai-Min Xu https://orcid.org/0000-0002-5230-4140

Chang-Ge Song https://orcid.org/0000-0001-5379-2353

Lu-Lu Shen https://orcid.org/0000-0002-4692-6488

Yi-Fei Sun https://orcid.org/0000-0003-3997-3662

Bao-Kai Cui https://orcid.org/0000-0003-3059-9344

### Data availability

The newly generated sequences have been uploaded to GenBank, and the sequences used for the multi-gene and individual-gene phylogenetic analyses are listed in Table 1, and those used for the ITS meta-analysis are provided in Suppl. material 1.

All alignments and resulting tree topologies conducted in this study have been deposited as follows: the multi-gene alignment and tree topology in Suppl. material 2; the treefile from the RAxML and MrBayes analyses of the multi-gene dataset in Suppl. materials 3, 4, respectively; the single-gene alignments and tree topologies for ITS, nLSU, and *tef1α* in Suppl. materials 5–7, respectively; the three single-gene trees with Bayesian support values annotated on the ML trees are provided in Suppl. material 8; the alignment and topology of the ITS meta-analysis in Suppl. material 9; the treefile from the RAxML analysis of the ITS meta-analysis in Suppl. material 10; and a vertical version of the ITS meta-analysis tree in Suppl. material 11.

## Supplementary materials

Supplementary material 1 Supplementary information 1 Data type xlsx Explanation note **table SS1**. ITS sequences excluded from the meta-analysis of *Hydnum* with various reasons. **table SS2**. ITS sequences remaining for the meta-analysis of *Hydnum*. **table S3**. The detailed information of ITS sequences used in meta-analysis of *Hydnum*, including the clustering relationship, acession number, locality, habitat and bootstraps. Bold names present the subgenera or clade. Asterisk (*) denote the information of type specimens. Asterisk (#) denote the information of environmental samples. https://binary.pensoft.net/file/1526243This dataset is made available under the Open Database License (http://opendatacommons.org/licenses/odbl/1.0/). The Open Database License (ODbL) is a license agreement intended to allow users to freely share, modify, and use this Dataset while maintaining this same freedom for others, provided that the original source and author(s) are credited. Yi-Hua Xu, Long Zeng, Tai-Min Xu, Chang-Ge Song, Lu-Lu Shen, Yi-Fei Sun, Bao-Kai Cui

Supplementary material 2 Supplementary information 2 Data type nex https://binary.pensoft.net/file/1526245This dataset is made available under the Open Database License (http://opendatacommons.org/licenses/odbl/1.0/). The Open Database License (ODbL) is a license agreement intended to allow users to freely share, modify, and use this Dataset while maintaining this same freedom for others, provided that the original source and author(s) are credited. Yi-Hua Xu, Long Zeng, Tai-Min Xu, Chang-Ge Song, Lu-Lu Shen, Yi-Fei Sun, Bao-Kai Cui

Supplementary material 3 Supplementary information 3 Data type treefile https://binary.pensoft.net/file/1526246This dataset is made available under the Open Database License (http://opendatacommons.org/licenses/odbl/1.0/). The Open Database License (ODbL) is a license agreement intended to allow users to freely share, modify, and use this Dataset while maintaining this same freedom for others, provided that the original source and author(s) are credited. Yi-Hua Xu, Long Zeng, Tai-Min Xu, Chang-Ge Song, Lu-Lu Shen, Yi-Fei Sun, Bao-Kai Cui

Supplementary material 4 Supplementary information 4 Data type tre https://binary.pensoft.net/file/1526247This dataset is made available under the Open Database License (http://opendatacommons.org/licenses/odbl/1.0/). The Open Database License (ODbL) is a license agreement intended to allow users to freely share, modify, and use this Dataset while maintaining this same freedom for others, provided that the original source and author(s) are credited. Yi-Hua Xu, Long Zeng, Tai-Min Xu, Chang-Ge Song, Lu-Lu Shen, Yi-Fei Sun, Bao-Kai Cui

Supplementary material 5 Supplementary information 5 Data type nex https://binary.pensoft.net/file/1526248This dataset is made available under the Open Database License (http://opendatacommons.org/licenses/odbl/1.0/). The Open Database License (ODbL) is a license agreement intended to allow users to freely share, modify, and use this Dataset while maintaining this same freedom for others, provided that the original source and author(s) are credited. Yi-Hua Xu, Long Zeng, Tai-Min Xu, Chang-Ge Song, Lu-Lu Shen, Yi-Fei Sun, Bao-Kai Cui

Supplementary material 6 Supplementary information 6 Data type nex https://binary.pensoft.net/file/1526249This dataset is made available under the Open Database License (http://opendatacommons.org/licenses/odbl/1.0/). The Open Database License (ODbL) is a license agreement intended to allow users to freely share, modify, and use this Dataset while maintaining this same freedom for others, provided that the original source and author(s) are credited. Yi-Hua Xu, Long Zeng, Tai-Min Xu, Chang-Ge Song, Lu-Lu Shen, Yi-Fei Sun, Bao-Kai Cui

Supplementary material 7 Supplementary information 7 Data type nex https://binary.pensoft.net/file/1526250This dataset is made available under the Open Database License (http://opendatacommons.org/licenses/odbl/1.0/). The Open Database License (ODbL) is a license agreement intended to allow users to freely share, modify, and use this Dataset while maintaining this same freedom for others, provided that the original source and author(s) are credited. Yi-Hua Xu, Long Zeng, Tai-Min Xu, Chang-Ge Song, Lu-Lu Shen, Yi-Fei Sun, Bao-Kai Cui

Supplementary material 8 Supplementary information 8 Data type pdf https://binary.pensoft.net/file/1526251This dataset is made available under the Open Database License (http://opendatacommons.org/licenses/odbl/1.0/). The Open Database License (ODbL) is a license agreement intended to allow users to freely share, modify, and use this Dataset while maintaining this same freedom for others, provided that the original source and author(s) are credited. Yi-Hua Xu, Long Zeng, Tai-Min Xu, Chang-Ge Song, Lu-Lu Shen, Yi-Fei Sun, Bao-Kai Cui

Supplementary material 9 Supplementary information 9 Data type nex https://binary.pensoft.net/file/1526252This dataset is made available under the Open Database License (http://opendatacommons.org/licenses/odbl/1.0/). The Open Database License (ODbL) is a license agreement intended to allow users to freely share, modify, and use this Dataset while maintaining this same freedom for others, provided that the original source and author(s) are credited. Yi-Hua Xu, Long Zeng, Tai-Min Xu, Chang-Ge Song, Lu-Lu Shen, Yi-Fei Sun, Bao-Kai Cui

Supplementary material 10 Supplementary information 10 Data type treefile https://binary.pensoft.net/file/1526253This dataset is made available under the Open Database License (http://opendatacommons.org/licenses/odbl/1.0/). The Open Database License (ODbL) is a license agreement intended to allow users to freely share, modify, and use this Dataset while maintaining this same freedom for others, provided that the original source and author(s) are credited. Yi-Hua Xu, Long Zeng, Tai-Min Xu, Chang-Ge Song, Lu-Lu Shen, Yi-Fei Sun, Bao-Kai Cui

Supplementary material 11 Supplementary information 11 Data type pdf https://binary.pensoft.net/file/1526254This dataset is made available under the Open Database License (http://opendatacommons.org/licenses/odbl/1.0/). The Open Database License (ODbL) is a license agreement intended to allow users to freely share, modify, and use this Dataset while maintaining this same freedom for others, provided that the original source and author(s) are credited. Yi-Hua Xu, Long Zeng, Tai-Min Xu, Chang-Ge Song, Lu-Lu Shen, Yi-Fei Sun, Bao-Kai Cui

## References

[B1] Ali M, Ghafoor A, Ghafoor A et al. (2024) *Hydnum khanspurense* sp. nov. and *H. berkeleyanum* from Pakistan. Phytotaxa 660: 161–170. 10.11646/phytotaxa.660.2.6

[B2] Badotti F, de Oliveira FS, Garcia CF et al. (2017) Effectiveness of ITS and sub-regions as DNA barcode markers for the identification of *Basidiomycota* (Fungi). BMC Microbiology 17: 1–12. 10.1186/s12866-017-0958-x PMC5322588 28228107

[B3] Baird RE, Khan SR (1986) The stipitate hydnums (*Thelephoraceae*) of Florida. Brittonia 38: 171–184. 10.2307/2807273

[B4] Baird RE, Wallace LE, Baker G et al. (2013) Stipitate hydnoid fungi of the temperate southeastern United States. Fungal Diversity 62: 41–114. 10.1007/s13225-013-0261-6

[B5] Baroni TJ, Swenie RA, Lacey L et al. (2025) *Hydnum (Cantharellales)* of the neotropics: four new species and new reports from Belize, Costa Rica, Dominican Republic, Mexico, and Panama. Mycological Progress 24: 13. 10.1007/s11557-024-02023-6

[B6] Buyck B, Duhem B, Das K et al. (2017) Fungal biodiversity profiles 21–30. Cryptogamie Mycologie 38: 101–146. 10.7872/crym/v38.iss1.2017.101

[B7] Cao T, Hu YP, Yu JR et al. (2021) A phylogenetic overview of the *Hydnaceae* (*Cantharellales*, *Basidiomycota*) with new taxa from China. Studies in Mycology 99: 100121–100121. 10.1016/j.simyco.2021.100121 PMC8717575 35035603

[B8] Chen JJ, Cui BK, He SH et al. (2016) Molecular phylogeny and global diversity of the remarkable genus *Bondarzewia* (*Basidiomycota*, *Russulales*). Mycologia 108: 697–708. 10.3852/14-216 27091389

[B9] Dizeci N, Karaca B, Onar O et al. (2021) The remarkable antibiofilm activity of the sweet tooth mushroom, *Hydnum repandum* (*Agaricomycetes*), displaying synergetic interactions with antibiotics. International. Journal of Medicinal Mushrooms 23: 45–60. 10.1615/IntJMedMushrooms.2021040148 34595891

[B10] Feng B, Wang XH, Ratkowsky D et al. (2016) Multilocus phylogenetic analyses reveal unexpected abundant diversity and significant disjunct distribution pattern of the hedgehog mushrooms (*Hydnum* L.). Scientific Reports 6: 25586. 10.1038/srep25586 PMC4858670 27151256

[B11] Garrab M, Edziri H, El Mokni R et al. (2019) Phenolic composition, antioxidant and anticholinesterase properties of the three mushrooms *Agaricus silvaticus* Schaeff., *Hydnum rufescens* Pers. and *Meripilus giganteus* (Pers.) Karst. in Tunisia. South African Journal of Botany 124: 359–363. 10.1016/j.sajb.2019.05.033

[B12] Grebenc T, Martín MP, Kraigher H (2009) Ribosomal ITS diversity among the European species of the genus “*Hydnum*” (*Hydnaceae*). In: Anales del Jardín Botánico de Madrid. Real Jardín Botánico, 121–132. 10.3989/ajbm.2221

[B13] Hall R (2002) Cenozoic geological and plate tectonic evolution of SE Asia and the SW Pacific: computer-based reconstructions, model and animations. Journal of Asian Earth Sciences 20: 353–431. 10.1016/S1367-9120(01)00069-4

[B14] Harrison KA (1964) New or little known North American stipitate hydnums. Canadian Journal of Botany 42: 1205–1233. 10.1139/b64-116

[B15] He XL, Peng WH, Wang D (2021) An illustration of important wild edible fungi in Sichuan. Science Press: Beijing, China, 206 pp.

[B16] Hofstetter V, Buyck B, Eyssartier G et al. (2019) The unbearable lightness of sequenced-based identification. Fungal Diversity 96: 243–284. 10.1007/s13225-019-00428-3

[B17] Justo A, Hood AW, Swenie RA et al. (2023) *Hydnum atlanticum*, a new species from Eastern North America. Fungal Systematics and Evolution 11: 63–70. 10.3114/fuse.2023.11.05 PMC10956580 38516386

[B18] Katoh K, Standley DM (2013) MAFFT multiple sequence alignment software version 7: improvements in performance and usability. Molecular Biology and Evolution 30: 772–780. 10.1093/molbev/mst010 PMC3603318 23329690

[B19] Kim JS, Lee W, Kim C et al. (2023) Unveiling the Diversity of *Hydnum* in the Republic of Korea with One New Species, *Hydnum paucispinum*. Mycobiology 51: 300–312. 10.1080/12298093.2023.2265137 PMC10621273 37929003

[B20] Kõljalg U, Nilsson RH, Abarenkov K et al. (2013) Towards a unified paradigm for sequence‐based identification of fungi. Molecular Ecology 22(21): 5271–5277. [Wiley Online Library, pp]. 10.1111/mec.12481 24112409

[B21] Kornerup A, Wanscher JH (1981) Taschenlexikon der Farben. 3. Auff. Muster-Schmidt Verlag, Göttingen, Germany, 242 pp.

[B22] Kranabetter J, Friesen J, Gamiet S et al. (2009) Epigeous fruiting bodies of ectomycorrhizal fungi as indicators of soil fertility and associated nitrogen status of boreal forests. Mycorrhiza 19: 535–548. 10.1007/s00572-009-0255-0 19449039

[B23] Linnaeus C (1753) Species Plantarum. Laurentius Salvius, 1–560 pp.

[B24] Liu S, Shen LL, Xu TM et al. (2023) Global diversity, molecular phylogeny and divergence times of the brown‑rot fungi within the *Polyporales*. Mycosphere 14: 1564–1664. 10.5943/mycosphere/14/1/18

[B25] Maas Geesteranus R (1960) Notes on hydnums. Persoonia 1: 341–384.

[B26] Márquez-Sanz R, Gorjón SP, Salcedo I et al. (2023) *Hydnum pallidum* Raddi, the correct name for *H. albidum* Peck in the sense of European authors and the recently described *H. reginae* Kibby, Liimat. & Niskanen. Journal of Fungi 9: 1141. 10.3390/jof9121141 PMC10744077 38132742

[B27] Nguyen LT, Schmidt HA, Von Haeseler A et al. (2015) IQ-TREE: a fast and effective stochastic algorithm for estimating maximum-likelihood phylogenies. Molecular Biology and Evolution 32: 268–274. 10.1093/molbev/msu300 PMC4271533 25371430

[B28] Nilsson RH, Ryberg M, Kristiansson E et al. (2006) Taxonomic reliability of DNA sequences in public sequence databases: a fungal perspective. PLoS ONE 1(1): e59. 10.1371/journal.pone.0000059 PMC1762357 17183689

[B29] Nilsson RH, Tedersoo L, Abarenkov K et al. (2012) Five simple guidelines for establishing basic authenticity and reliability of newly generated fungal ITS sequences. MycoKeys 4: 37–63. 10.3897/mycokeys.4.3606

[B30] Niskanen T, Liimatainen K, Nuytinck J et al. (2018) Identifying and naming the currently known diversity of the genus *Hydnum*, with an emphasis on European and North American taxa. Mycologia 110: 890–918. 10.1080/00275514.2018.1477004 30215579

[B31] Olariaga I, Grebenc T, Salcedo I et al. (2012) Two new species of *Hydnum* with ovoid basidiospores: *H. ovoideisporum* and *H. vesterholtii*. Mycologia 104: 1443–1455. 10.3852/11-378 22684286

[B32] Ostrow H, Beenken L (2004) *Hydnum ellipsosporum* spec. nov. (*Basidiomycetes*, *Cantharellales*)–ein Doppelgänger von *Hydnum rufescens* Fr. Zeitschrift fuer Mykologie 70: 137–156.

[B33] Qin HZ, Liao YT, Zhang YZ et al. (2024) A contribution to the knowledge of *Hydnum* (*Hydnaceae*, *Cantharellales*) in China, introducing a new taxon and amending descriptions of five known species. Diversity Basel 16: 166. 10.3390/d16030166

[B34] Schoch CL, Seifert KA, Huhndorf S et al. (2012) Nuclear ribosomal internal transcribed spacer (ITS) region as a universal DNA barcode marker for Fungi. Proceedings of the national academy of Sciences 109: 6241–6246. 10.1073/pnas.1117018109 PMC3341068 22454494

[B35] Su LJ, Yu TJ, Xue R et al. (2024) New contributions on species diversity of genus *Hydnum* and *Lentaria* s.l. in China. Journal of Fungi 10: 824. 10.3390/jof10120824 PMC11678670 39728320

[B36] Song CG, Xu TM, Xu YH et al. (2025) Systematic revision, molecular phylogeny and divergence times of *Thelephorales (Basidiomycota)*. Mycosphere 16: 296–422. 10.5943/mycosphere/16/1/5

[B37] Sugawara R, Sotome K, Maekawa N et al. (2021) Mycorrhizal synthesis, morpho-anatomical characterization of mycorrhizae, and evaluation of mycorrhiza-forming ability of *Hydnum albidum*-like species using monokaryotic and dikaryotic cultures. Mycorrhiza 31: 349–359. 10.1007/s00572-021-01024-7 33616720

[B38] Sugawara R, Maekawa N, Sotome K et al. (2022) Systematic revision of *Hydnum* species in Japan. Mycologia 114: 413–452. 10.1080/00275514.2021.2024407 35394899

[B39] Summerbell RC, Moore MK, Starink-Willemse M et al. (2007) ITS barcodes for *Trichophyton tonsurans* and *T. equinum*. *Sabouraudia* 45: 193–200. 10.1080/13693780601087614 17464840

[B40] Sun YF, Xing JH, He XL et al. (2022) Species diversity, systematic revision and molecular phylogeny of *Ganodermataceae* (*Polyporales*, *Basidiomycota*) with an emphasis on Chinese collections. Studies in Mycology 101: 287–415. 10.3114/sim.2022.101.05 PMC9365044 36059897

[B41] Swenie RA, Baroni TJ, Matheny PB (2018) Six new species and reports of *Hydnum (Cantharellales)* from eastern North America. MycoKeys 42: 35–72. 10.3897/mycokeys.42.27369 PMC6286385 30564052

[B42] Swenie RA, Looney BP, Ke YH et al. (2024) PacBio high‐throughput multi-locus sequencing reveals high genetic diversity in mushroom‐forming fungi. Molecular Ecology Resources 24: e13885. 10.1111/1755-0998.13885 37902171

[B43] Tuo YL, Wang LB, Li XF et al. (2025) New contributions to the species diversity of the genus *Hydnum* (*Hydnaceae*, *Cantharellales*) in China: four new taxa and newly recorded species. Journal of Fungi 11: 431. 10.3390/jof11060431 PMC12194605 40558943

[B44] Vilgalys R, Hester M (1990) Rapid genetic identification and mapping of enzymatically amplified ribosomal DNA from several *Cryptococcus* species. Journal of bacteriology 172: 4238–4246. 10.1128/jb.172.8.4238-4246.1990 PMC213247 2376561

[B45] Vizzini A, Picillo B, Ercole E et al. (2013) Detecting the variability of *Hydnum ovoideisporum* (*Agaricomycetes*, *Cantharellales*) on the basis of Italian collections, and *H. magnorufescens* sp. nov. Mycosphere 4: 32–44. 10.5943/mycosphere/4/1/2

[B46] Wang XH, Das K, Horman J et al. (2018) Fungal biodiversity profiles 51-60. Cryptogamie Mycologie 39: 211–257. 10.7872/crym/v39.iss2.2018.211

[B47] White TJ, Bruns T, Lee S et al. (1990) Amplification and direct sequencing of fungal ribosomal RNA genes for phylogenetics. PCR Protocols: A Guide to Methods and Applications 18: 315–322. 10.1016/B978-0-12-372180-8.50042-1

[B48] Yanaga K, Sotome K, Ushijima S et al. (2015) *Hydnum* species producing whitish basidiomata in Japan. Mycoscience 56: 434–442. 10.1016/j.myc.2015.01.001

[B49] Zhang M, Wang CQ, Bai HF et al. (2024) A contribution to the phylogeny and taxonomy of *Hydnum* (*Cantharellales*, *Basidiomycota*) from China. Journal of Fungi 10: 98. 10.3390/jof10020098 PMC10889965 38392770

