# Supplementary material for: Multi-gene phylogeny and taxonomy of Hydnum (Hydnaceae, Cantharellales) including a global phylogeny of ITS sequences for the genus
Source: IMA Fungus. 2026 Feb 3;17:e172544. doi: 10.3897/imafungus.17.172544 (PMC12892097; doi:10.3897/imafungus.17.172544)
Supplement: Supplementary material 8 — Supplementary information 8 [file imafungus-17-e172544-s008.pdf]

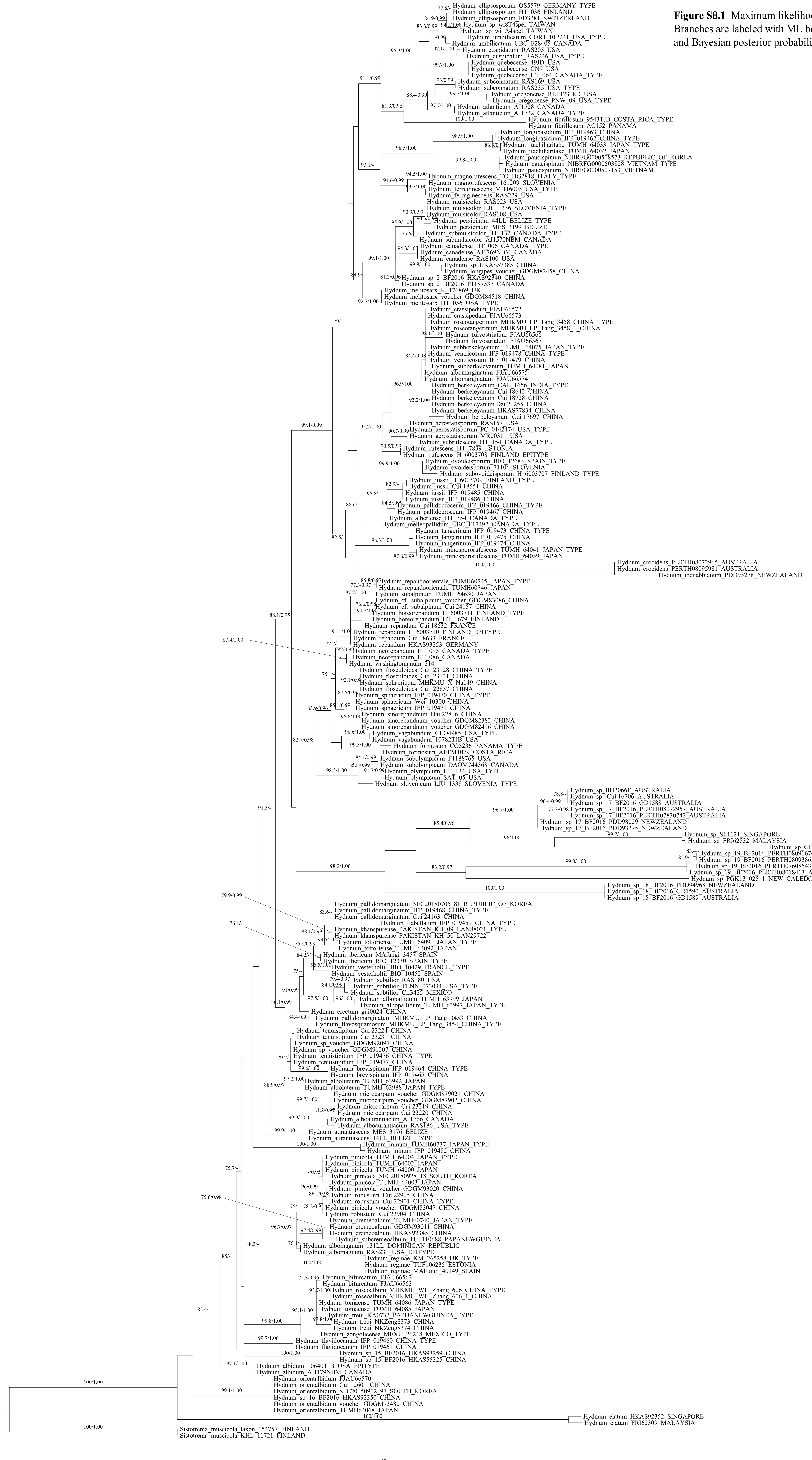

Figure S8.1 Maximum likelihood tree of the Hydnum based on ITS. Branches are labeled with ML bootstrap values higher than 75%, and Bayesian posterior probabilities higher than 0.95 respectively.

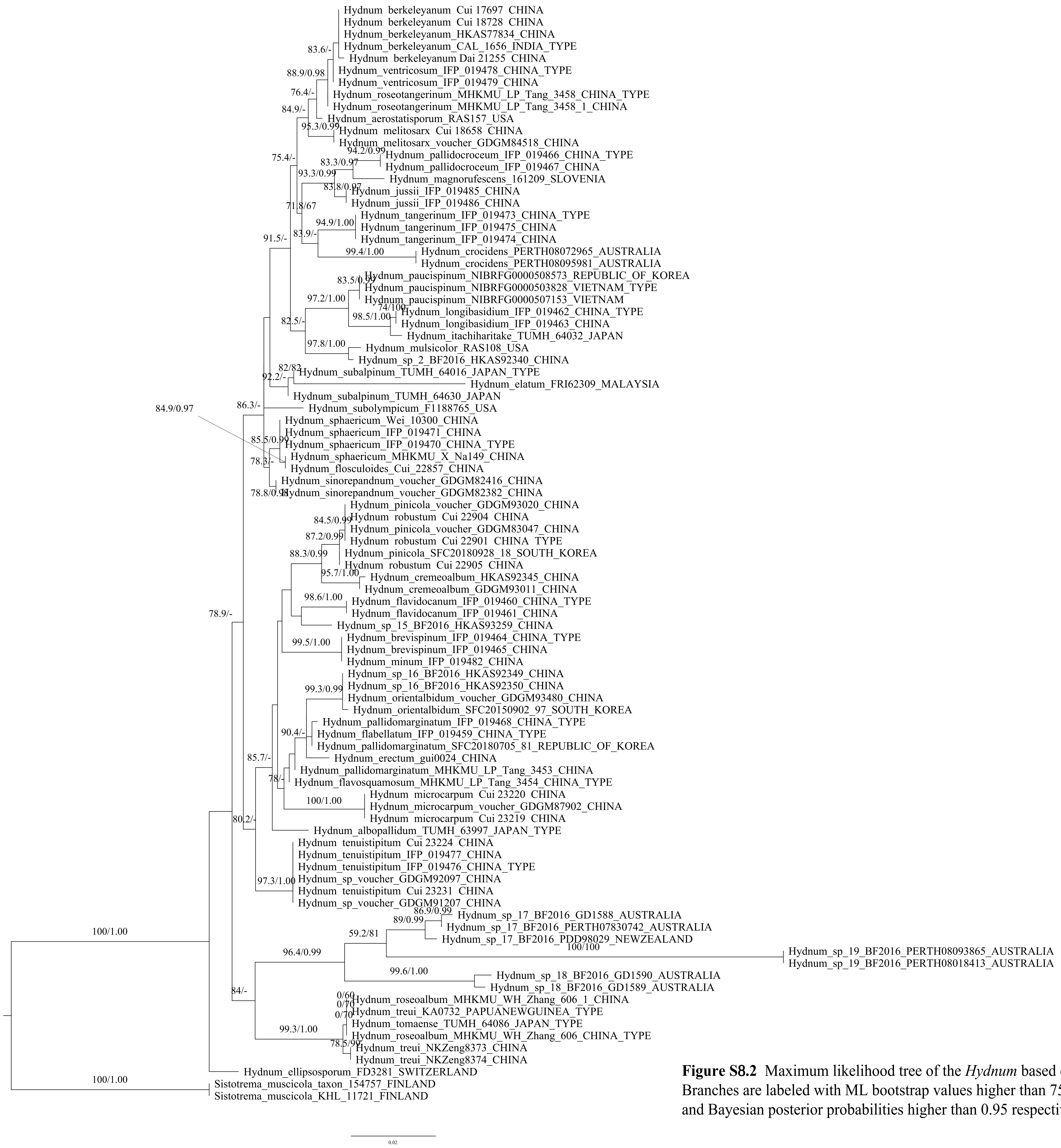

Figure S8.2 Maximum likelihood tree of the *Hydnum* based on **nLSU**. Branches are labeled with ML bootstrap values higher than 75%, and Bayesian posterior probabilities higher than 0.95 respectively.

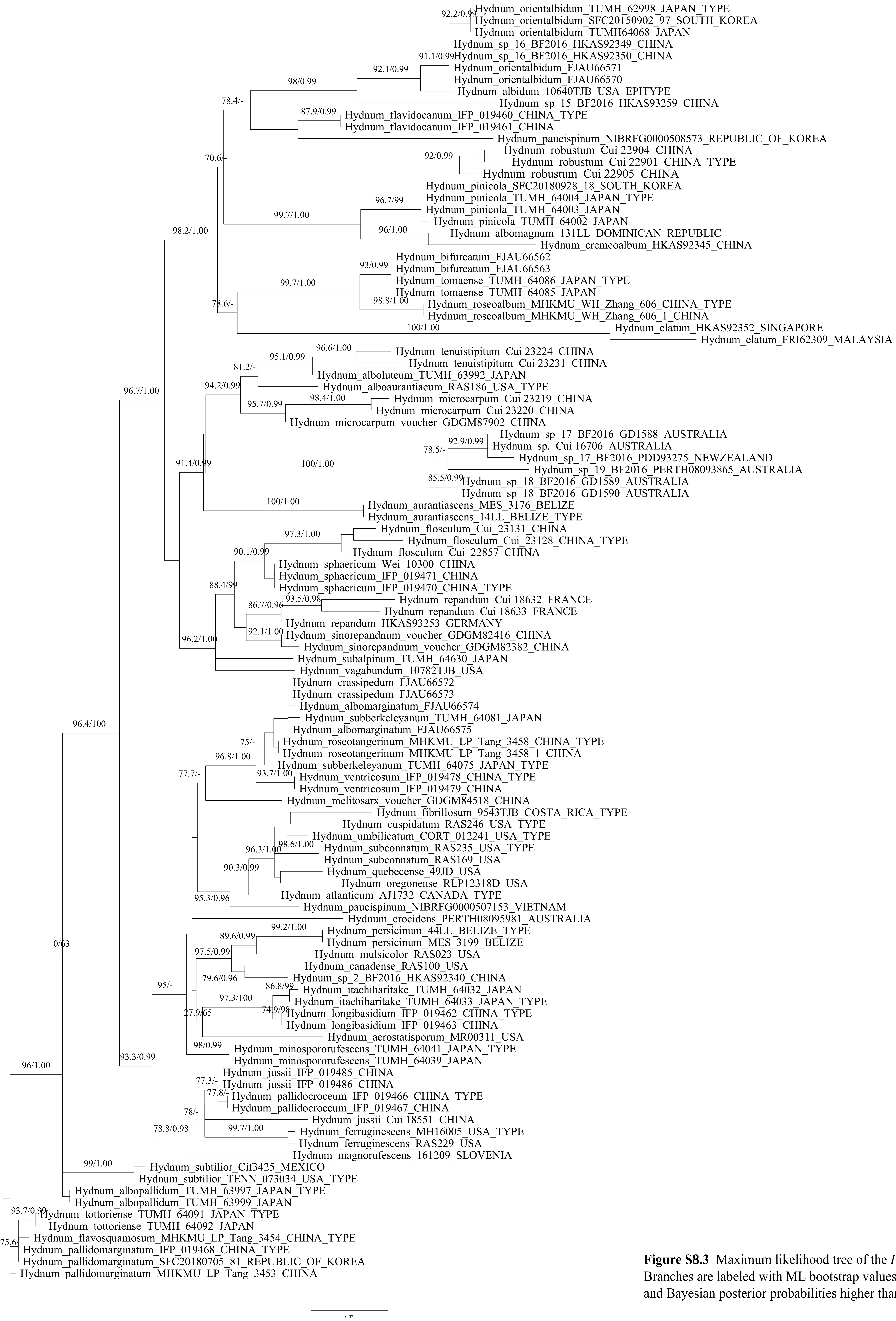

Figure S8.3 Maximum likelihood tree of the *Hydnum* based on ***tef1α***. Branches are labeled with ML bootstrap values higher than 75%, and Bayesian posterior probabilities higher than 0.95 respectively.
