# Supplementary material for: Multi-gene phylogeny and taxonomy of Hydnum (Hydnaceae, Cantharellales) including a global phylogeny of ITS sequences for the genus
Source: IMA Fungus. 2026 Feb 3;17:e172544. doi: 10.3897/imafungus.17.172544 (PMC12892097; doi:10.3897/imafungus.17.172544)
Supplement: Supplementary material 11 — Supplementary information 11 [file imafungus-17-e172544-s011.pdf]

Figure S11 Maximum likelihood tree of *Hydnum* based on ITS sequences.

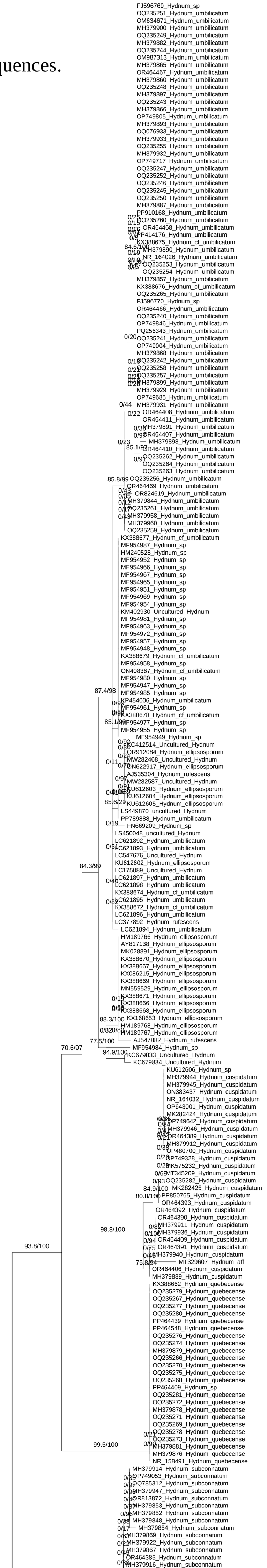

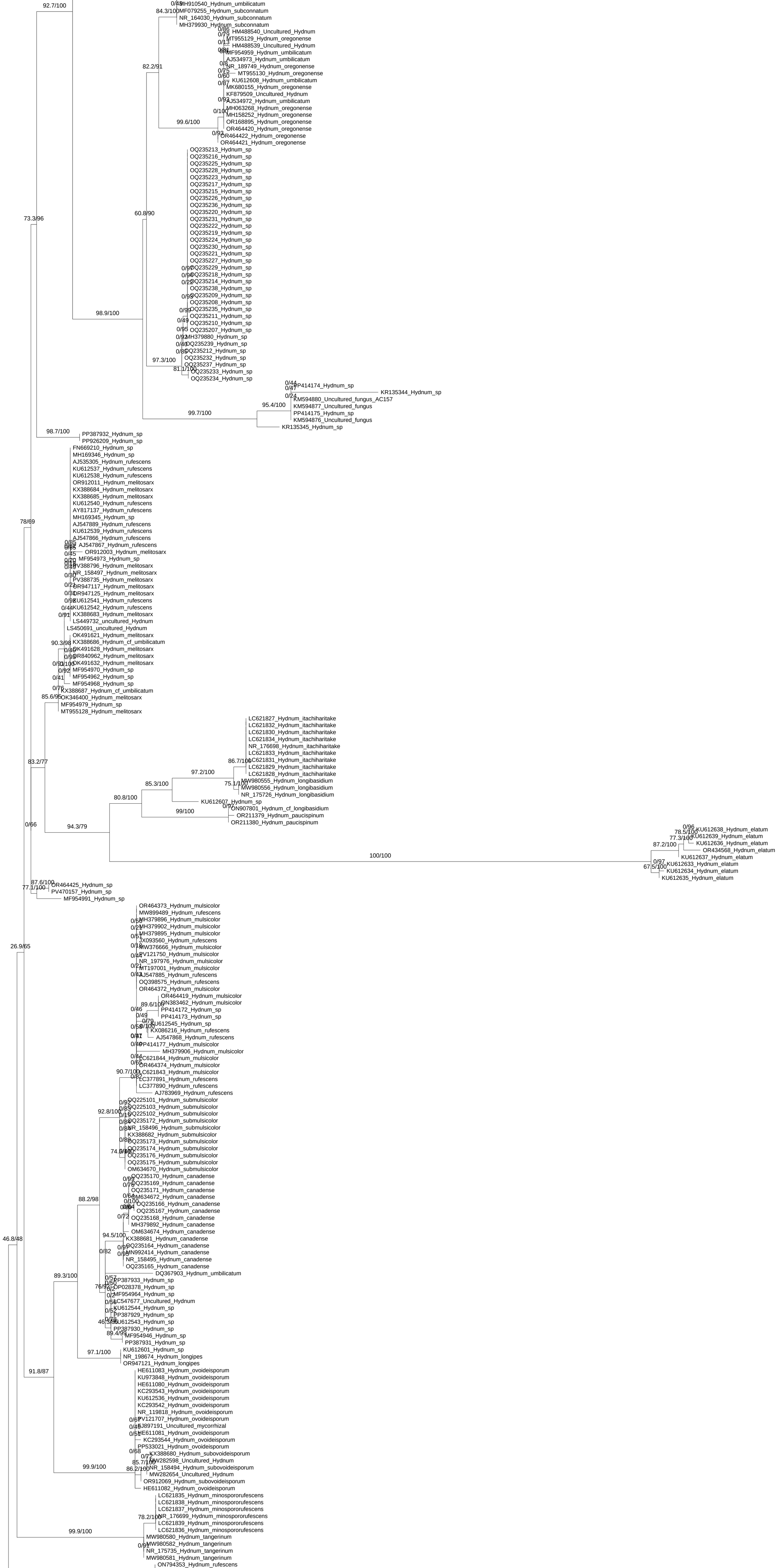
92.7/100
MH910540_Hydnum_umbilicatum
MF079255_Hydnum_subconnatum
NR_164030_Hydnum_subconnatum
MH379930_Hydnum_subconnatum
84.3/100
HM488540_Uncultured_Hydnum
MT955129_Hydnum_oregonense
HM488539_Uncultured_Hydnum
MF954959_Hydnum_umbilicatum
AJ534973_Hydnum_umbilicatum
NR_189749_Hydnum_oregonense
MT955130_Hydnum_oregonense
KU612608_Hydnum_umbilicatum
MK680155_Hydnum_oregonense
KF879509_Uncultured_Hydnum
AJ534972_Hydnum_umbilicatum
MH063268_Hydnum_oregonense
MH158252_Hydnum_oregonense
OR168895_Hydnum_oregonense
OR464420_Hydnum_oregonense
OR464422_Hydnum_oregonense
OR464421_Hydnum_oregonense
82.2/91
99.6/100
OQ235213_Hydnum_sp
OQ235216_Hydnum_sp
OQ235225_Hydnum_sp
OQ235228_Hydnum_sp
OQ235223_Hydnum_sp
OQ235217_Hydnum_sp
OQ235215_Hydnum_sp
OQ235226_Hydnum_sp
OQ235236_Hydnum_sp
OQ235220_Hydnum_sp
OQ235231_Hydnum_sp
OQ235222_Hydnum_sp
OQ235219_Hydnum_sp
OQ235224_Hydnum_sp
OQ235230_Hydnum_sp
OQ235221_Hydnum_sp
OQ235227_Hydnum_sp
OQ235229_Hydnum_sp
OQ235218_Hydnum_sp
OQ235214_Hydnum_sp
OQ235238_Hydnum_sp
OQ235209_Hydnum_sp
OQ235208_Hydnum_sp
OQ235235_Hydnum_sp
OQ235211_Hydnum_sp
OQ235210_Hydnum_sp
OQ235207_Hydnum_sp
MH379880_Hydnum_sp
OQ235239_Hydnum_sp
OQ235212_Hydnum_sp
OQ235232_Hydnum_sp
OQ235237_Hydnum_sp
OQ235233_Hydnum_sp
OQ235234_Hydnum_sp
73.3/96
60.8/90
98.9/100
97.3/100
81.1/100
PP414174_Hydnum_sp
KR135344_Hydnum_sp
KM594880_Uncultured_fungus_AC157
KM594877_Uncultured_fungus
PP414175_Hydnum_sp
KM594876_Uncultured_fungus
KR135345_Hydnum_sp
95.4/100
99.7/100
98.7/100
PP387932_Hydnum_sp
PP926209_Hydnum_sp
FN669210_Hydnum_sp
MH169346_Hydnum_sp
AJ535305_Hydnum_rufescens
KU612537_Hydnum_rufescens
KU612538_Hydnum_rufescens
OR912011_Hydnum_melitosarx
KX388684_Hydnum_melitosarx
KX388685_Hydnum_melitosarx
KU612540_Hydnum_rufescens
AY817137_Hydnum_rufescens
MH169345_Hydnum_sp
AJ547889_Hydnum_rufescens
KU612539_Hydnum_rufescens
AJ547866_Hydnum_rufescens
AJ547867_Hydnum_rufescens
OR912003_Hydnum_melitosarx
MF954973_Hydnum_sp
PV388796_Hydnum_melitosarx
NR_158497_Hydnum_melitosarx
PV388735_Hydnum_melitosarx
OR947117_Hydnum_melitosarx
OR947125_Hydnum_melitosarx
KU612541_Hydnum_rufescens
KU612542_Hydnum_rufescens
KX388683_Hydnum_melitosarx
LS449732_uncultured_Hydnum
LS450691_uncultured_Hydnum
OK491621_Hydnum_melitosarx
KX388686_Hydnum_cf_umbilicatum
OK491628_Hydnum_melitosarx
OR840962_Hydnum_melitosarx
OK491632_Hydnum_melitosarx
MF954970_Hydnum_sp
MF954962_Hydnum_sp
MF954968_Hydnum_sp
KX388687_Hydnum_cf_umbilicatum
OK346400_Hydnum_melitosarx
MF954979_Hydnum_sp
MT955128_Hydnum_melitosarx
78/69
90.3/98
85.6/95
LC621827_Hydnum_itachiharitake
LC621832_Hydnum_itachiharitake
LC621830_Hydnum_itachiharitake
LC621834_Hydnum_itachiharitake
NR_176698_Hydnum_itachiharitake
LC621833_Hydnum_itachiharitake
LC621831_Hydnum_itachiharitake
LC621829_Hydnum_itachiharitake
LC621828_Hydnum_itachiharitake
MW980555_Hydnum_longibasidium
MW980556_Hydnum_longibasidium
NR_175726_Hydnum_longibasidium
KU612607_Hydnum_sp
ON907801_Hydnum_cf_longibasidium
OR211379_Hydnum_paucispinum
OR211380_Hydnum_paucispinum
83.2/77
86.7/100
97.2/100
75.1/100
85.3/100
80.8/100
99/100
0/66
94.3/79
KU612638_Hydnum_elatum
KU612639_Hydnum_elatum
KU612636_Hydnum_elatum
OR434568_Hydnum_elatum
KU612637_Hydnum_elatum
KU612633_Hydnum_elatum
KU612634_Hydnum_elatum
KU612635_Hydnum_elatum
100/100
78.5/100
77.3/100
87.2/100
67.5/100
OR464425_Hydnum_sp
PV470157_Hydnum_sp
MF954991_Hydnum_sp
87.6/100
77.1/100
26.9/65
OR464373_Hydnum_mulsicolor
MW899489_Hydnum_rufescens
MH379896_Hydnum_mulsicolor
MH379902_Hydnum_mulsicolor
MH379895_Hydnum_mulsicolor
JX093560_Hydnum_rufescens
MW376666_Hydnum_mulsicolor
PV121750_Hydnum_mulsicolor
NR_197976_Hydnum_mulsicolor
MT197001_Hydnum_mulsicolor
AJ547885_Hydnum_rufescens
OQ398575_Hydnum_rufescens
OR464372_Hydnum_mulsicolor
OR464419_Hydnum_mulsicolor
ON383462_Hydnum_mulsicolor
PP414172_Hydnum_sp
PP414173_Hydnum_sp
KU612545_Hydnum_sp
KX086216_Hydnum_rufescens
AJ547868_Hydnum_rufescens
PP414177_Hydnum_mulsicolor
MH379906_Hydnum_mulsicolor
LC621844_Hydnum_mulsicolor
OR464374_Hydnum_mulsicolor
LC621843_Hydnum_mulsicolor
LC377891_Hydnum_rufescens
LC377890_Hydnum_rufescens
AJ783969_Hydnum_rufescens
89.6/100
90.7/100
OQ225101_Hydnum_submulsicolor
OQ225103_Hydnum_submulsicolor
OQ225102_Hydnum_submulsicolor
OQ235172_Hydnum_submulsicolor
NR_158496_Hydnum_submulsicolor
KX388682_Hydnum_submulsicolor
OQ235173_Hydnum_submulsicolor
OQ235174_Hydnum_submulsicolor
OQ235176_Hydnum_submulsicolor
OQ235175_Hydnum_submulsicolor
OM634670_Hydnum_submulsicolor
92.8/100
OQ235170_Hydnum_canadense
OQ235169_Hydnum_canadense
OQ235171_Hydnum_canadense
OM634672_Hydnum_canadense
OQ235166_Hydnum_canadense
OQ235167_Hydnum_canadense
OQ235168_Hydnum_canadense
MH379892_Hydnum_canadense
OM634674_Hydnum_canadense
KX388681_Hydnum_canadense
OQ235164_Hydnum_canadense
MN992414_Hydnum_canadense
NR_158495_Hydnum_canadense
OQ235165_Hydnum_canadense
88.2/98
94.5/100
DQ367903_Hydnum_umbilicatum
PP387933_Hydnum_sp
OP028378_Hydnum_sp
MF954964_Hydnum_sp
LC547677_Uncultured_Hydnum
KU612544_Hydnum_sp
PP387929_Hydnum_sp
KU612543_Hydnum_sp
PP387930_Hydnum_sp
MF954946_Hydnum_sp
PP387931_Hydnum_sp
KU612601_Hydnum_sp
NR_198674_Hydnum_longipes
OR947121_Hydnum_longipes
89.3/100
89.4/99
97.1/100
46.8/48
91.8/87
HE611083_Hydnum_ovoideisporum
KU973848_Hydnum_ovoideisporum
HE611080_Hydnum_ovoideisporum
KC293543_Hydnum_ovoideisporum
KU612536_Hydnum_ovoideisporum
KC293542_Hydnum_ovoideisporum
NR_119818_Hydnum_ovoideisporum
PV121707_Hydnum_ovoideisporum
EJ897191_Uncultured_mycorrhizal
HE611081_Hydnum_ovoideisporum
KC293544_Hydnum_ovoideisporum
PP533021_Hydnum_ovoideisporum
KX388680_Hydnum_subovoideisporum
MW282598_Uncultured_Hydnum
NR_158494_Hydnum_subovoideisporum
MW282654_Uncultured_Hydnum
OR912069_Hydnum_subovoideisporum
HE611082_Hydnum_ovoideisporum
85.7/100
86.2/100
99.9/100
LC621835_Hydnum_minospororufescens
LC621838_Hydnum_minospororufescens
LC621837_Hydnum_minospororufescens
NR_176699_Hydnum_minospororufescens
LC621839_Hydnum_minospororufescens
LC621836_Hydnum_minospororufescens
MW980580_Hydnum_tangerinum
MW980582_Hydnum_tangerinum
NR_175735_Hydnum_tangerinum
MW980581_Hydnum_tangerinum
ON794353_Hydnum_rufescens
78.2/100
99.9/100

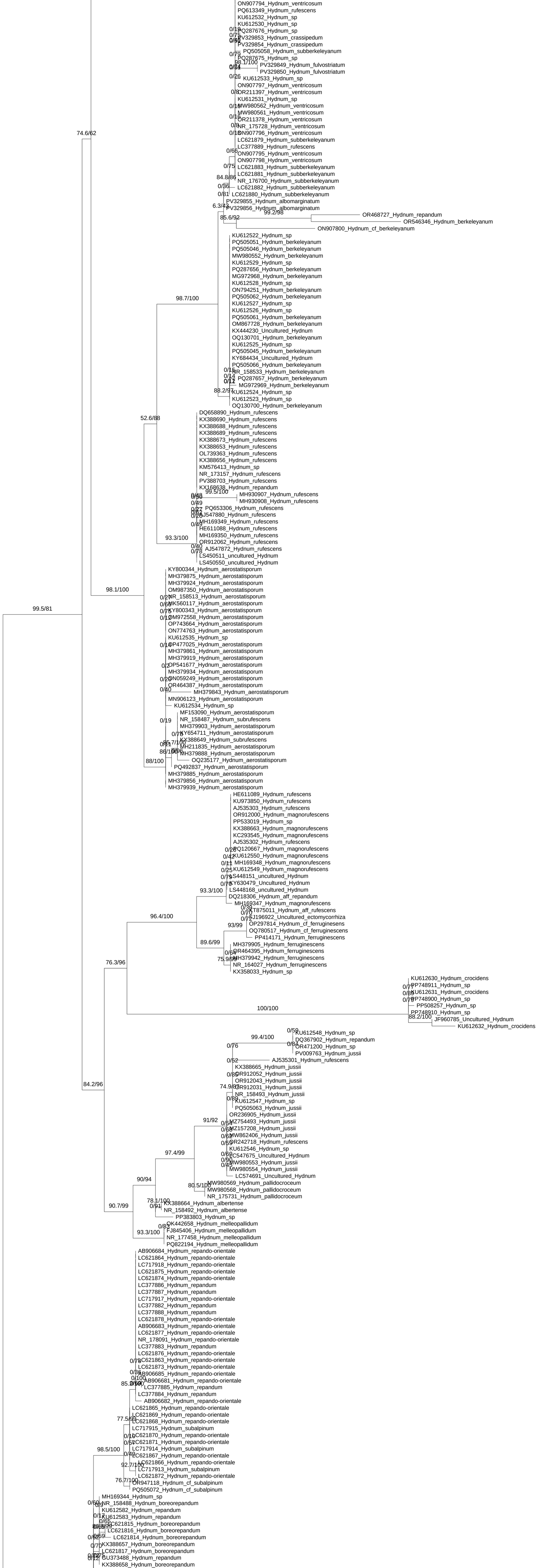
ON907794_Hydnum_ventricosum
PQ613349_Hydnum_rufescens
KU612532_Hydnum_sp
KU612530_Hydnum_sp
PQ287676_Hydnum_sp
PV329853_Hydnum_crassipedum
PV329854_Hydnum_crassipedum
PQ505058_Hydnum_subberkeleyanum
PQ287675_Hydnum_sp
98.1/100
PV329849_Hydnum_fulvostriatum
PV329850_Hydnum_fulvostriatum
KU612533_Hydnum_sp
ON907797_Hydnum_ventricosum
OR211397_Hydnum_ventricosum
KU612531_Hydnum_sp
MW980562_Hydnum_ventricosum
MW980561_Hydnum_ventricosum
OR211378_Hydnum_ventricosum
NR_175728_Hydnum_ventricosum
ON907796_Hydnum_ventricosum
74.6/62
LC621879_Hydnum_subberkeleyanum
LC377889_Hydnum_rufescens
ON907795_Hydnum_ventricosum
ON907798_Hydnum_ventricosum
LC621883_Hydnum_subberkeleyanum
LC621881_Hydnum_subberkeleyanum
84.8/86
NR_176700_Hydnum_subberkeleyanum
LC621882_Hydnum_subberkeleyanum
LC621880_Hydnum_subberkeleyanum
PV329855_Hydnum_albomarginatum
PV329856_Hydnum_albomarginatum
99.2/98
OR468727_Hydnum_repandum
85.6/92
OR546346_Hydnum_berkeleyanum
ON907800_Hydnum_cf_berkeleyanum
KU612522_Hydnum_sp
PQ505051_Hydnum_berkeleyanum
PQ505046_Hydnum_berkeleyanum
MW980552_Hydnum_berkeleyanum
KU612529_Hydnum_sp
PQ287656_Hydnum_berkeleyanum
MG972968_Hydnum_berkeleyanum
KU612528_Hydnum_sp
ON794251_Hydnum_berkeleyanum
98.7/100
PQ505062_Hydnum_berkeleyanum
KU612527_Hydnum_sp
KU612526_Hydnum_sp
PQ505061_Hydnum_berkeleyanum
OM867728_Hydnum_berkeleyanum
KX444230_Uncultured_Hydnum
OQ130701_Hydnum_berkeleyanum
KU612525_Hydnum_sp
PQ505045_Hydnum_berkeleyanum
KY684434_Uncultured_Hydnum
PQ505066_Hydnum_berkeleyanum
NR_158533_Hydnum_berkeleyanum
PQ287657_Hydnum_berkeleyanum
MG972969_Hydnum_berkeleyanum
KU612524_Hydnum_sp
KU612523_Hydnum_sp
OQ130700_Hydnum_berkeleyanum
DQ658890_Hydnum_rufescens
KX388690_Hydnum_rufescens
52.6/88
KX388688_Hydnum_rufescens
KX388689_Hydnum_rufescens
KX388673_Hydnum_rufescens
KX388653_Hydnum_rufescens
OL739363_Hydnum_rufescens
KX388656_Hydnum_rufescens
KM576413_Hydnum_sp
NR_173157_Hydnum_rufescens
PV388703_Hydnum_rufescens
KX168638_Hydnum_repandum
99.5/100
MH930907_Hydnum_rufescens
MH930908_Hydnum_rufescens
PQ653306_Hydnum_rufescens
AJ547880_Hydnum_rufescens
MH169349_Hydnum_rufescens
HE611088_Hydnum_rufescens
93.3/100
MH169350_Hydnum_rufescens
OR912062_Hydnum_rufescens
AJ547872_Hydnum_rufescens
LS450511_uncultured_Hydnum
LS450550_uncultured_Hydnum
KY800344_Hydnum_aerostatisporum
MH379875_Hydnum_aerostatisporum
MH379924_Hydnum_aerostatisporum
98.1/100
OM987350_Hydnum_aerostatisporum
NR_158513_Hydnum_aerostatisporum
99.5/81
MK560117_Hydnum_aerostatisporum
KY800343_Hydnum_aerostatisporum
OM972558_Hydnum_aerostatisporum
OP743664_Hydnum_aerostatisporum
ON774763_Hydnum_aerostatisporum
KU612535_Hydnum_sp
OP477025_Hydnum_aerostatisporum
MH379861_Hydnum_aerostatisporum
MH379919_Hydnum_aerostatisporum
OP541677_Hydnum_aerostatisporum
MH379934_Hydnum_aerostatisporum
ON059249_Hydnum_aerostatisporum
OR464387_Hydnum_aerostatisporum
MH379843_Hydnum_aerostatisporum
MN906123_Hydnum_aerostatisporum
KU612534_Hydnum_sp
MF153090_Hydnum_aerostatisporum
NR_158487_Hydnum_subrufescens
MH379903_Hydnum_aerostatisporum
KY654711_Hydnum_aerostatisporum
KX388649_Hydnum_subrufescens
MH211835_Hydnum_aerostatisporum
MH379888_Hydnum_aerostatisporum
88/100
OQ235177_Hydnum_aerostatisporum
PQ492837_Hydnum_aerostatisporum
MH379885_Hydnum_aerostatisporum
MH379856_Hydnum_aerostatisporum
MH379939_Hydnum_aerostatisporum
HE611089_Hydnum_rufescens
KU973850_Hydnum_rufescens
AJ535303_Hydnum_rufescens
OR912000_Hydnum_magnorufescens
PP533019_Hydnum_sp
KX388663_Hydnum_magnorufescens
KC293545_Hydnum_magnorufescens
AJ535302_Hydnum_rufescens
PQ120667_Hydnum_magnorufescens
KU612550_Hydnum_magnorufescens
MH169348_Hydnum_magnorufescens
KU612549_Hydnum_magnorufescens
LS448151_uncultured_Hydnum
KY630479_Uncultured_Hydnum
93.3/100
LS448168_uncultured_Hydnum
DQ218306_Hydnum_aff_repandum
MH169347_Hydnum_magnorufescens
96.4/100
KT875011_Hydnum_aff_rufescens
EJ196922_Uncultured_ectomycorrhiza
OP297814_Hydnum_cf_ferruginescens
93/99
OQ780517_Hydnum_cf_ferruginescens
PP414171_Hydnum_ferruginescens
89.6/99
MH379905_Hydnum_ferruginescens
OR464395_Hydnum_ferruginescens
76.3/96
MH379942_Hydnum_ferruginescens
NR_164027_Hydnum_ferruginescens
KX358033_Hydnum_sp
KU612630_Hydnum_crocidens
PP748911_Hydnum_sp
KU612631_Hydnum_crocidens
PP748900_Hydnum_sp
100/100
PP508257_Hydnum_sp
PP748910_Hydnum_sp
88.2/100
JF960785_Uncultured_Hydnum
KU612632_Hydnum_crocidens
KU612548_Hydnum_sp
99.4/100
DQ367902_Hydnum_repandum
OR471200_Hydnum_sp
PV009763_Hydnum_jussii
AJ535301_Hydnum_rufescens
KX388665_Hydnum_jussii
84.2/96
OR912052_Hydnum_jussii
OR912043_Hydnum_jussii
OR912031_Hydnum_jussii
NR_158493_Hydnum_jussii
KU612547_Hydnum_sp
PQ505063_Hydnum_jussii
91/92
OR236905_Hydnum_jussii
MZ754493_Hydnum_jussii
MZ157208_Hydnum_jussii
MW862406_Hydnum_jussii
OR242718_Hydnum_rufescens
KU612546_Hydnum_sp
97.4/99
LC547675_Uncultured_Hydnum
MW980553_Hydnum_jussii
MW980554_Hydnum_jussii
LC574691_Uncultured_Hydnum
90/94
80.5/100
MW980569_Hydnum_pallidocroceum
MW980568_Hydnum_pallidocroceum
NR_175731_Hydnum_pallidocroceum
78.1/100
KX388664_Hydnum_albertense
90.7/99
NR_158492_Hydnum_albertense
PP383803_Hydnum_sp
OK442658_Hydnum_melleopallidum
FJ845406_Hydnum_melleopallidum
93.3/100
NR_177458_Hydnum_melleopallidum
PQ822194_Hydnum_melleopallidum
AB906684_Hydnum_repando-orientale
LC621864_Hydnum_repando-orientale
LC717918_Hydnum_repando-orientale
LC621875_Hydnum_repando-orientale
LC621874_Hydnum_repando-orientale
LC377886_Hydnum_repandum
LC377887_Hydnum_repandum
LC717917_Hydnum_repando-orientale
LC377882_Hydnum_repandum
LC377888_Hydnum_repandum
LC621878_Hydnum_repando-orientale
AB906683_Hydnum_repando-orientale
LC621877_Hydnum_repando-orientale
NR_178091_Hydnum_repando-orientale
LC377883_Hydnum_repandum
LC621876_Hydnum_repando-orientale
LC621863_Hydnum_repando-orientale
LC621873_Hydnum_repando-orientale
AB906685_Hydnum_repando-orientale
AB906681_Hydnum_repando-orientale
LC377885_Hydnum_repandum
LC377884_Hydnum_repandum
AB906682_Hydnum_repando-orientale
LC621865_Hydnum_repando-orientale
LC621869_Hydnum_repando-orientale
LC621868_Hydnum_repando-orientale
LC717915_Hydnum_subalpinum
LC621870_Hydnum_repando-orientale
LC621871_Hydnum_repando-orientale
LC717914_Hydnum_subalpinum
98.5/100
LC621867_Hydnum_repando-orientale
LC621866_Hydnum_repando-orientale
LC717913_Hydnum_subalpinum
LC621872_Hydnum_repando-orientale
76.7/100
OR947118_Hydnum_cf_subalpinum
PQ505072_Hydnum_cf_subalpinum
MH169344_Hydnum_sp
NR_158488_Hydnum_boreorepandum
KU612582_Hydnum_repandum
KU612583_Hydnum_repandum
LC621815_Hydnum_boreorepandum
LC621816_Hydnum_boreorepandum
LC621814_Hydnum_boreorepandum
KX388657_Hydnum_boreorepandum
LC621817_Hydnum_boreorepandum
GU373488_Hydnum_repandum
KX388658_Hydnum_boreorepandum

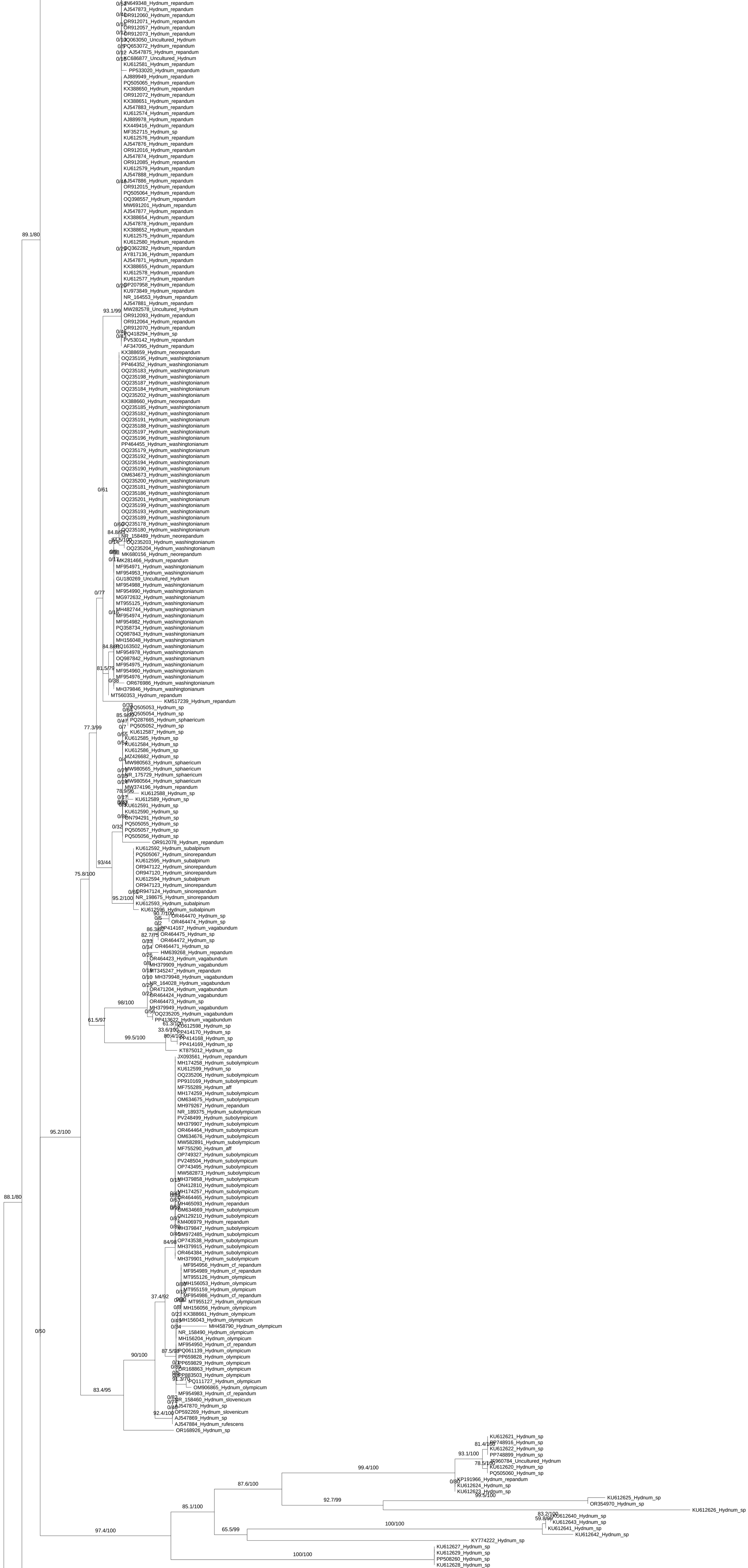

KU612589_Hydnum_repandum
AJ547873_Hydnum_repandum
OR912060_Hydnum_repandum
OR912071_Hydnum_repandum
OR912057_Hydnum_repandum
OR912073_Hydnum_repandum
OQ063050_Uncultured_Hydnum
PQ653072_Hydnum_repandum
AJ547875_Hydnum_repandum
LC686877_Uncultured_Hydnum
KU612581_Hydnum_repandum
PP533020_Hydnum_repandum
AJ889949_Hydnum_repandum
PQ505065_Hydnum_repandum
KX388650_Hydnum_repandum
OR912072_Hydnum_repandum
KX388651_Hydnum_repandum
AJ547883_Hydnum_repandum
KU612574_Hydnum_repandum
AJ889978_Hydnum_repandum
KX449416_Hydnum_repandum
MF352715_Hydnum_sp
KU612576_Hydnum_repandum
AJ547876_Hydnum_repandum
OR912016_Hydnum_repandum
AJ547874_Hydnum_repandum
OR912085_Hydnum_repandum
KU612579_Hydnum_repandum
AJ547888_Hydnum_repandum
AJ547886_Hydnum_repandum
OR912015_Hydnum_repandum
PQ505064_Hydnum_repandum
OQ398557_Hydnum_repandum
MW691201_Hydnum_repandum
AJ547877_Hydnum_repandum
KX388654_Hydnum_repandum
AJ547878_Hydnum_repandum
KX388652_Hydnum_repandum
KU612575_Hydnum_repandum
KU612580_Hydnum_repandum
OQ362282_Hydnum_repandum
AY817136_Hydnum_repandum
AJ547871_Hydnum_repandum
KX388655_Hydnum_repandum
KU612578_Hydnum_repandum
KU612577_Hydnum_repandum
OP207958_Hydnum_repandum
KU973849_Hydnum_repandum
NR_164553_Hydnum_repandum
AJ547881_Hydnum_repandum
MW282578_Uncultured_Hydnum
OR912093_Hydnum_repandum
OR912064_Hydnum_repandum
OR912070_Hydnum_repandum
PQ418294_Hydnum_sp
PV530142_Hydnum_repandum
AF347095_Hydnum_repandum
KX388659_Hydnum_neorepandum
OQ235195_Hydnum_washingtonianum
PP464352_Hydnum_washingtonianum
OQ235183_Hydnum_washingtonianum
OQ235198_Hydnum_washingtonianum
OQ235187_Hydnum_washingtonianum
OQ235184_Hydnum_washingtonianum
OQ235202_Hydnum_washingtonianum
KX388660_Hydnum_neorepandum
OQ235185_Hydnum_washingtonianum
OQ235182_Hydnum_washingtonianum
OQ235191_Hydnum_washingtonianum
OQ235188_Hydnum_washingtonianum
OQ235197_Hydnum_washingtonianum
OQ235196_Hydnum_washingtonianum
PP464455_Hydnum_washingtonianum
OQ235179_Hydnum_washingtonianum
OQ235192_Hydnum_washingtonianum
OQ235194_Hydnum_washingtonianum
OQ235190_Hydnum_washingtonianum
OM634673_Hydnum_washingtonianum
OQ235200_Hydnum_washingtonianum
OQ235181_Hydnum_washingtonianum
OQ235186_Hydnum_washingtonianum
OQ235201_Hydnum_washingtonianum
OQ235199_Hydnum_washingtonianum
OQ235193_Hydnum_washingtonianum
OQ235189_Hydnum_washingtonianum
OQ235178_Hydnum_washingtonianum
OQ235180_Hydnum_washingtonianum
NR_158489_Hydnum_neorepandum
OQ235203_Hydnum_washingtonianum
OQ235204_Hydnum_washingtonianum
MK680156_Hydnum_neorepandum
MK281466_Hydnum_repandum
MF954971_Hydnum_washingtonianum
MF954953_Hydnum_washingtonianum
GU180269_Uncultured_Hydnum
MF954988_Hydnum_washingtonianum
MF954990_Hydnum_washingtonianum
MG972632_Hydnum_washingtonianum
MT955125_Hydnum_washingtonianum
MH482744_Hydnum_washingtonianum
MF954974_Hydnum_washingtonianum
MF954982_Hydnum_washingtonianum
PQ358734_Hydnum_washingtonianum
OQ987843_Hydnum_washingtonianum
MH156048_Hydnum_washingtonianum
PQ163502_Hydnum_washingtonianum
MF954978_Hydnum_washingtonianum
OQ987842_Hydnum_washingtonianum
MF954975_Hydnum_washingtonianum
MF954960_Hydnum_washingtonianum
MF954976_Hydnum_washingtonianum
OR676986_Hydnum_washingtonianum
MH379846_Hydnum_washingtonianum
MT560353_Hydnum_repandum
KM517239_Hydnum_repandum
PQ505053_Hydnum_sp
PQ505054_Hydnum_sp
PQ287665_Hydnum_sphaericum
PQ505052_Hydnum_sp
KU612587_Hydnum_sp
KU612585_Hydnum_sp
KU612584_Hydnum_sp
KU612586_Hydnum_sp
MZ426682_Hydnum_sp
MW980563_Hydnum_sphaericum
MW980565_Hydnum_sphaericum
NR_175729_Hydnum_sphaericum
MW980564_Hydnum_sphaericum
MW374196_Hydnum_repandum
KU612588_Hydnum_sp
KU612589_Hydnum_sp
KU612591_Hydnum_sp
KU612590_Hydnum_sp
ON794291_Hydnum_sp
PQ505055_Hydnum_sp
PQ505057_Hydnum_sp
PQ505056_Hydnum_sp
OR912078_Hydnum_repandum
KU612592_Hydnum_subalpinum
PQ505067_Hydnum_sinorepandum
KU612595_Hydnum_subalpinum
OR947122_Hydnum_sinorepandum
OR947120_Hydnum_sinorepandum
KU612594_Hydnum_subalpinum
OR947123_Hydnum_sinorepandum
OR947124_Hydnum_sinorepandum
NR_198675_Hydnum_sinorepandum
KU612593_Hydnum_subalpinum
KU612596_Hydnum_subalpinum
OR464470_Hydnum_sp
OR464474_Hydnum_sp
PP414167_Hydnum_vagabundum
OR464475_Hydnum_sp
OR464472_Hydnum_sp
OR464471_Hydnum_sp
HM639268_Hydnum_repandum
OR464423_Hydnum_vagabundum
MH379909_Hydnum_vagabundum
MT345247_Hydnum_repandum
MH379948_Hydnum_vagabundum
NR_164028_Hydnum_vagabundum
OR471204_Hydnum_vagabundum
OR464424_Hydnum_vagabundum
OR464473_Hydnum_sp
MH379949_Hydnum_vagabundum
OQ235205_Hydnum_vagabundum
PP413622_Hydnum_vagabundum
KU612598_Hydnum_sp
PP414170_Hydnum_sp
PP414168_Hydnum_sp
PP414169_Hydnum_sp
KT875012_Hydnum_sp
JX093561_Hydnum_repandum
MH174258_Hydnum_subolympicum
KU612599_Hydnum_sp
OQ235206_Hydnum_subolympicum
PP910169_Hydnum_subolympicum
MF755289_Hydnum_aff
MH174259_Hydnum_subolympicum
OM634675_Hydnum_subolympicum
MH979267_Hydnum_repandum
NR_189375_Hydnum_subolympicum
PV248499_Hydnum_subolympicum
MH379907_Hydnum_subolympicum
OR464464_Hydnum_subolympicum
OM634676_Hydnum_subolympicum
MW582891_Hydnum_subolympicum
MF755290_Hydnum_aff
OP749327_Hydnum_subolympicum
PV248504_Hydnum_subolympicum
OP743495_Hydnum_subolympicum
MW582873_Hydnum_subolympicum
MH379858_Hydnum_subolympicum
ON412810_Hydnum_subolympicum
MH174257_Hydnum_subolympicum
OR464465_Hydnum_subolympicum
MH465093_Hydnum_repandum
OM634669_Hydnum_subolympicum
ON129210_Hydnum_subolympicum
KM406979_Hydnum_repandum
MH379847_Hydnum_subolympicum
OM972485_Hydnum_subolympicum
OP743538_Hydnum_subolympicum
MH379915_Hydnum_subolympicum
OR464384_Hydnum_subolympicum
MH379901_Hydnum_subolympicum
MF954956_Hydnum_cf_repandum
MF954989_Hydnum_cf_repandum
MT955126_Hydnum_olympicum
MH156053_Hydnum_olympicum
MT955159_Hydnum_olympicum
MF954986_Hydnum_cf_repandum
MT955127_Hydnum_olympicum
MH156056_Hydnum_olympicum
KX388661_Hydnum_olympicum
MH156043_Hydnum_olympicum
MH458790_Hydnum_olympicum
NR_158490_Hydnum_olympicum
MH156204_Hydnum_olympicum
MF954950_Hydnum_cf_repandum
PQ061139_Hydnum_olympicum
PP659828_Hydnum_olympicum
PP659829_Hydnum_olympicum
OR168863_Hydnum_olympicum
PP883503_Hydnum_olympicum
PQ111727_Hydnum_olympicum
OM906865_Hydnum_olympicum
MF954983_Hydnum_cf_repandum
NR_158460_Hydnum_slovenicum
AJ547870_Hydnum_sp
OP592269_Hydnum_slovenicum
AJ547869_Hydnum_sp
AJ547884_Hydnum_rufescens
OR168926_Hydnum_sp
KU612621_Hydnum_sp
PP748916_Hydnum_sp
KU612622_Hydnum_sp
PP748899_Hydnum_sp
JF960784_Uncultured_Hydnum
KU612620_Hydnum_sp
PQ505060_Hydnum_sp
KP191966_Hydnum_repandum
KU612624_Hydnum_sp
KU612623_Hydnum_sp
KU612625_Hydnum_sp
OR354970_Hydnum_sp
KU612626_Hydnum_sp
KU612640_Hydnum_sp
KU612643_Hydnum_sp
KU612641_Hydnum_sp
KU612642_Hydnum_sp
KY774222_Hydnum_sp
KU612627_Hydnum_sp
KU612629_Hydnum_sp
PP508260_Hydnum_sp
KU612628_Hydnum_sp
89.1/80
93.1/99
0/61
84.8/69
0/77
84.8/80
81.5/79
0/38
77.3/99
93/44
95.2/100
75.8/100
98/100
61.5/97
99.5/100
95.2/100
88.1/80
84/98
37.4/92
90/100
87.5/98
92.4/100
83.4/95
0/50
99.4/100
93.1/100
81.4/100
78.5/100
87.6/100
92.7/99
99.5/100
85.1/100
100/100
83.2/100
59.8/99
65.5/99
97.4/100
100/100

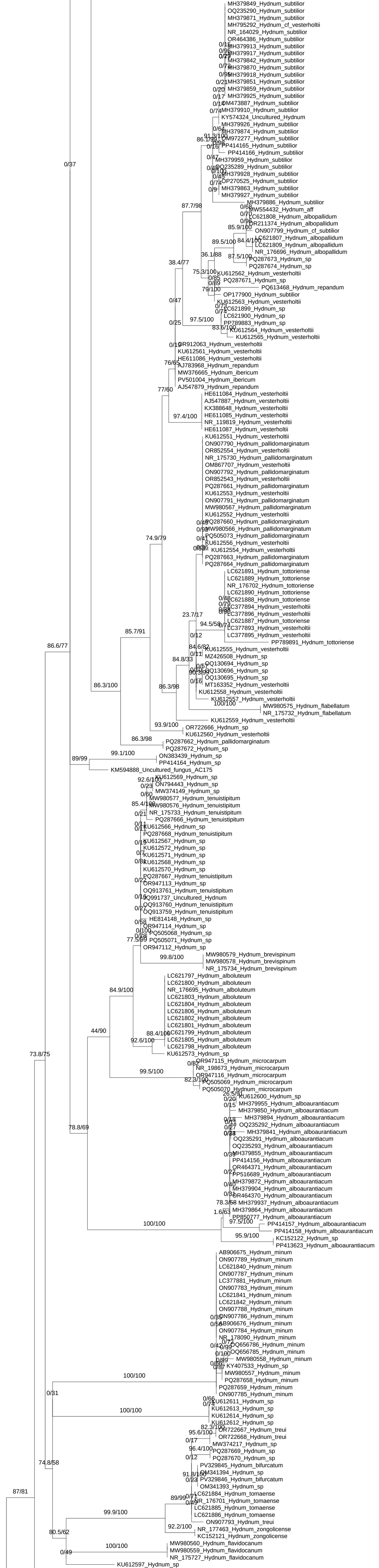
MH379849_Hydnum_subtilior
OQ235290_Hydnum_subtilior
MH379871_Hydnum_subtilior
MH795292_Hydnum_cf_vesterholtii
NR_164029_Hydnum_subtilior
OR464386_Hydnum_subtilior
MH379913_Hydnum_subtilior
MH379917_Hydnum_subtilior
MH379842_Hydnum_subtilior
MH379870_Hydnum_subtilior
MH379918_Hydnum_subtilior
MH379851_Hydnum_subtilior
MH379859_Hydnum_subtilior
MH379925_Hydnum_subtilior
OM473887_Hydnum_subtilior
MH379910_Hydnum_subtilior
KY574324_Uncultured_Hydnum
MH379926_Hydnum_subtilior
MH379874_Hydnum_subtilior
OM972277_Hydnum_subtilior
PP414165_Hydnum_subtilior
PP414166_Hydnum_subtilior
MH379959_Hydnum_subtilior
OQ235289_Hydnum_subtilior
MH379928_Hydnum_subtilior
OP270525_Hydnum_subtilior
MH379863_Hydnum_subtilior
MH379927_Hydnum_subtilior
MH379886_Hydnum_subtilior
MW554432_Hydnum_aff
LC621808_Hydnum_albopallidum
OR211374_Hydnum_albopallidum
ON907799_Hydnum_cf_subtilior
LC621807_Hydnum_albopallidum
LC621809_Hydnum_albopallidum
NR_176696_Hydnum_albopallidum
PQ287673_Hydnum_sp
PQ287674_Hydnum_sp
KU612562_Hydnum_vesterholtii
PQ287671_Hydnum_sp
PQ613468_Hydnum_repandum
OP177900_Hydnum_subtilior
KU612563_Hydnum_vesterholtii
LC621899_Hydnum_sp
LC621900_Hydnum_sp
PP789883_Hydnum_sp
KU612564_Hydnum_vesterholtii
KU612565_Hydnum_vesterholtii
OR912063_Hydnum_vesterholtii
KU612561_Hydnum_vesterholtii
HE611086_Hydnum_vesterholtii
AJ783968_Hydnum_repandum
MW376665_Hydnum_ibericum
PV501004_Hydnum_ibericum
AJ547879_Hydnum_repandum
HE611084_Hydnum_vesterholtii
AJ547887_Hydnum_versterholtii
KX388648_Hydnum_vesterholtii
HE611085_Hydnum_vesterholtii
NR_119819_Hydnum_vesterholtii
HE611087_Hydnum_vesterholtii
KU612551_Hydnum_vesterholtii
ON907790_Hydnum_pallidomarginatum
OR852554_Hydnum_vesterholtii
NR_175730_Hydnum_pallidomarginatum
OM867707_Hydnum_vesterholtii
ON907792_Hydnum_pallidomarginatum
OR852543_Hydnum_vesterholtii
PQ287661_Hydnum_pallidomarginatum
KU612553_Hydnum_vesterholtii
ON907791_Hydnum_pallidomarginatum
MW980567_Hydnum_pallidomarginatum
KU612552_Hydnum_vesterholtii
PQ287660_Hydnum_pallidomarginatum
MW980566_Hydnum_pallidomarginatum
PQ505073_Hydnum_pallidomarginatum
KU612556_Hydnum_vesterholtii
KU612554_Hydnum_vesterholtii
PQ287663_Hydnum_pallidomarginatum
PQ287664_Hydnum_pallidomarginatum
LC621891_Hydnum_tottoriense
LC621889_Hydnum_tottoriense
NR_176702_Hydnum_tottoriense
LC621890_Hydnum_tottoriense
LC621888_Hydnum_tottoriense
LC377894_Hydnum_vesterholtii
LC377896_Hydnum_vesterholtii
LC621887_Hydnum_tottoriense
LC377893_Hydnum_vesterholtii
LC377895_Hydnum_vesterholtii
PP789891_Hydnum_tottoriense
KU612555_Hydnum_vesterholtii
MZ426508_Hydnum_sp
OQ130694_Hydnum_sp
OQ130696_Hydnum_sp
OQ130695_Hydnum_sp
MT163352_Hydnum_vesterholtii
KU612558_Hydnum_vesterholtii
KU612557_Hydnum_vesterholtii
MW980575_Hydnum_flabellatum
NR_175732_Hydnum_flabellatum
KU612559_Hydnum_vesterholtii
OR722666_Hydnum_sp
KU612560_Hydnum_vesterholtii
PQ287662_Hydnum_pallidomarginatum
PQ287672_Hydnum_sp
ON383439_Hydnum_sp
PP414164_Hydnum_sp
KM594888_Uncultured_fungus_AC175
KU612569_Hydnum_sp
ON794443_Hydnum_sp
MW374149_Hydnum_sp
MW980577_Hydnum_tenuistipitum
MW980576_Hydnum_tenuistipitum
NR_175733_Hydnum_tenuistipitum
PQ287666_Hydnum_tenuistipitum
KU612566_Hydnum_sp
PQ287668_Hydnum_tenuistipitum
KU612567_Hydnum_sp
KU612572_Hydnum_sp
KU612571_Hydnum_sp
KU612568_Hydnum_sp
KU612570_Hydnum_sp
PQ287667_Hydnum_tenuistipitum
OR947113_Hydnum_sp
OQ913761_Hydnum_tenuistipitum
OQ991737_Uncultured_Hydnum
OQ913760_Hydnum_tenuistipitum
OQ913759_Hydnum_tenuistipitum
HE814148_Hydnum_sp
OR947114_Hydnum_sp
PQ505068_Hydnum_sp
PQ505071_Hydnum_sp
OR947112_Hydnum_sp
MW980579_Hydnum_brevispinum
MW980578_Hydnum_brevispinum
NR_175734_Hydnum_brevispinum
LC621797_Hydnum_alboluteum
LC621800_Hydnum_alboluteum
NR_176695_Hydnum_alboluteum
LC621803_Hydnum_alboluteum
LC621804_Hydnum_alboluteum
LC621806_Hydnum_alboluteum
LC621802_Hydnum_alboluteum
LC621801_Hydnum_alboluteum
LC621799_Hydnum_alboluteum
LC621805_Hydnum_alboluteum
LC621798_Hydnum_alboluteum
KU612573_Hydnum_sp
OR947115_Hydnum_microcarpum
NR_198673_Hydnum_microcarpum
OR947116_Hydnum_microcarpum
PQ505069_Hydnum_microcarpum
PQ505070_Hydnum_microcarpum
KU612600_Hydnum_sp
MH379955_Hydnum_alboaurantiacum
MH379850_Hydnum_alboaurantiacum
MH379894_Hydnum_alboaurantiacum
OQ235292_Hydnum_alboaurantiacum
MH379841_Hydnum_alboaurantiacum
OQ235291_Hydnum_alboaurantiacum
OQ235293_Hydnum_alboaurantiacum
MH379855_Hydnum_alboaurantiacum
PP414156_Hydnum_alboaurantiacum
OR464371_Hydnum_alboaurantiacum
PP516689_Hydnum_alboaurantiacum
MH379872_Hydnum_alboaurantiacum
MH379904_Hydnum_alboaurantiacum
OR464370_Hydnum_alboaurantiacum
MH379937_Hydnum_alboaurantiacum
MH379864_Hydnum_alboaurantiacum
PP850777_Hydnum_alboaurantiacum
PP414157_Hydnum_alboaurantiacum
PP414158_Hydnum_alboaurantiacum
KC152122_Hydnum_sp
PP413623_Hydnum_alboaurantiacum
AB906675_Hydnum_minum
ON907789_Hydnum_minum
LC621840_Hydnum_minum
ON907787_Hydnum_minum
LC377881_Hydnum_minum
ON907783_Hydnum_minum
LC621841_Hydnum_minum
LC621842_Hydnum_minum
ON907788_Hydnum_minum
ON907786_Hydnum_minum
AB906676_Hydnum_minum
ON907784_Hydnum_minum
NR_178090_Hydnum_minum
OQ656786_Hydnum_minum
OQ656785_Hydnum_minum
MW980558_Hydnum_minum
KY407533_Hydnum_sp
MW980557_Hydnum_minum
PQ287658_Hydnum_minum
PQ287659_Hydnum_minum
ON907785_Hydnum_minum
KU612611_Hydnum_sp
KU612613_Hydnum_sp
KU612614_Hydnum_sp
KU612612_Hydnum_sp
OR722667_Hydnum_treui
OR722668_Hydnum_treui
MW374217_Hydnum_sp
PQ287669_Hydnum_sp
PQ287670_Hydnum_sp
PV329845_Hydnum_bifurcatum
OM341394_Hydnum_sp
PV329846_Hydnum_bifurcatum
OM341393_Hydnum_sp
LC621884_Hydnum_tomaense
NR_176701_Hydnum_tomaense
LC621885_Hydnum_tomaense
LC621886_Hydnum_tomaense
ON907793_Hydnum_treui
NR_177463_Hydnum_zongolicense
KC152121_Hydnum_zongolicense
MW980560_Hydnum_flavidocanum
MW980559_Hydnum_flavidocanum
NR_175727_Hydnum_flavidocanum
KU612597_Hydnum_sp
0/37
87.7/98
38.4/77
0/47
0/25
97.5/100
76/65
77/60
97.4/100
74.9/79
85.7/91
86.6/77
86.3/100
86.3/98
93.9/100
86.3/98
89/99
99.1/100
99.8/100
84.9/100
44/90
92.6/100
99.5/100
73.8/75
78.8/69
100/100
95.9/100
100/100
0/31
100/100
74.8/58
87/81
99.9/100
80.5/62
92.2/100
100/100
0/49

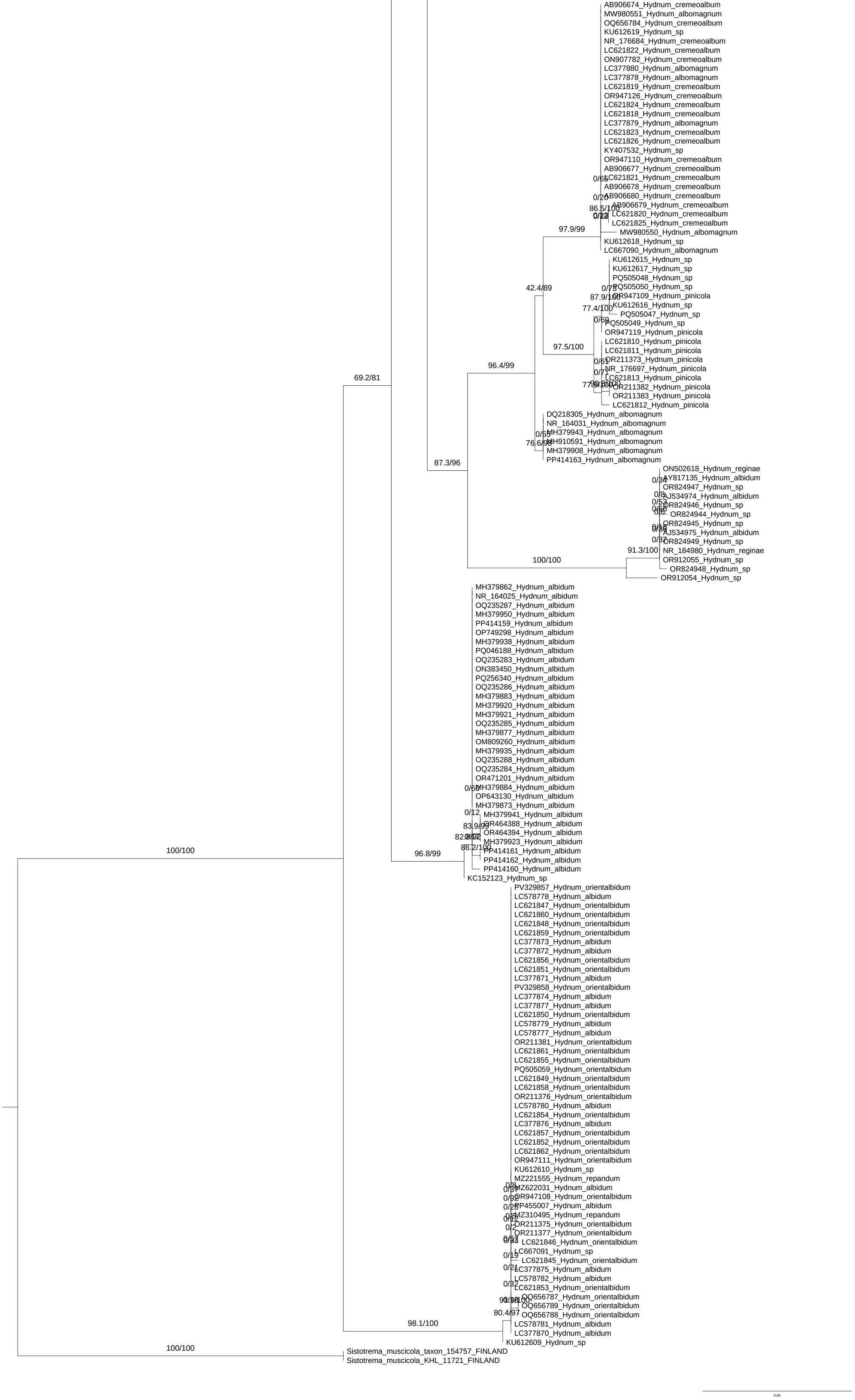

AB906674_Hydnum_cremeoalbum
MW980551_Hydnum_albomagnum
OQ656784_Hydnum_cremeoalbum
KU612619_Hydnum_sp
NR_176684_Hydnum_cremeoalbum
LC621822_Hydnum_cremeoalbum
ON907782_Hydnum_cremeoalbum
LC377880_Hydnum_albomagnum
LC377878_Hydnum_albomagnum
LC621819_Hydnum_cremeoalbum
OR947126_Hydnum_cremeoalbum
LC621824_Hydnum_cremeoalbum
LC621818_Hydnum_cremeoalbum
LC377879_Hydnum_albomagnum
LC621823_Hydnum_cremeoalbum
LC621826_Hydnum_cremeoalbum
KY407532_Hydnum_sp
OR947110_Hydnum_cremeoalbum
AB906677_Hydnum_cremeoalbum
LC621821_Hydnum_cremeoalbum
AB906678_Hydnum_cremeoalbum
AB906680_Hydnum_cremeoalbum
AB906679_Hydnum_cremeoalbum
LC621820_Hydnum_cremeoalbum
LC621825_Hydnum_cremeoalbum
MW980550_Hydnum_albomagnum
KU612618_Hydnum_sp
LC667090_Hydnum_albomagnum
KU612615_Hydnum_sp
KU612617_Hydnum_sp
PQ505048_Hydnum_sp
PQ505050_Hydnum_sp
OR947109_Hydnum_pinicola
KU612616_Hydnum_sp
PQ505047_Hydnum_sp
PQ505049_Hydnum_sp
OR947119_Hydnum_pinicola
LC621810_Hydnum_pinicola
LC621811_Hydnum_pinicola
OR211373_Hydnum_pinicola
NR_176697_Hydnum_pinicola
LC621813_Hydnum_pinicola
OR211382_Hydnum_pinicola
OR211383_Hydnum_pinicola
LC621812_Hydnum_pinicola
DQ218305_Hydnum_albomagnum
NR_164031_Hydnum_albomagnum
MH379943_Hydnum_albomagnum
MH910591_Hydnum_albomagnum
MH379908_Hydnum_albomagnum
PP414163_Hydnum_albomagnum
ON502618_Hydnum_reginae
AY817135_Hydnum_albidum
OR824947_Hydnum_sp
AJ534974_Hydnum_albidum
OR824946_Hydnum_sp
OR824944_Hydnum_sp
OR824945_Hydnum_sp
AJ534975_Hydnum_albidum
OR824949_Hydnum_sp
NR_184980_Hydnum_reginae
OR912055_Hydnum_sp
OR824948_Hydnum_sp
OR912054_Hydnum_sp
MH379862_Hydnum_albidum
NR_164025_Hydnum_albidum
OQ235287_Hydnum_albidum
MH379950_Hydnum_albidum
PP414159_Hydnum_albidum
OP749298_Hydnum_albidum
MH379938_Hydnum_albidum
PQ046188_Hydnum_albidum
OQ235283_Hydnum_albidum
ON383450_Hydnum_albidum
PQ256340_Hydnum_albidum
OQ235286_Hydnum_albidum
MH379883_Hydnum_albidum
MH379920_Hydnum_albidum
MH379921_Hydnum_albidum
OQ235285_Hydnum_albidum
MH379877_Hydnum_albidum
OM809260_Hydnum_albidum
MH379935_Hydnum_albidum
OQ235288_Hydnum_albidum
OQ235284_Hydnum_albidum
OR471201_Hydnum_albidum
MH379884_Hydnum_albidum
OP643130_Hydnum_albidum
MH379873_Hydnum_albidum
MH379941_Hydnum_albidum
OR464388_Hydnum_albidum
OR464394_Hydnum_albidum
MH379923_Hydnum_albidum
PP414161_Hydnum_albidum
PP414162_Hydnum_albidum
PP414160_Hydnum_albidum
KC152123_Hydnum_sp
PV329857_Hydnum_orientalbidum
LC578778_Hydnum_albidum
LC621847_Hydnum_orientalbidum
LC621860_Hydnum_orientalbidum
LC621848_Hydnum_orientalbidum
LC621859_Hydnum_orientalbidum
LC377873_Hydnum_albidum
LC377872_Hydnum_albidum
LC621856_Hydnum_orientalbidum
LC621851_Hydnum_orientalbidum
LC377871_Hydnum_albidum
PV329858_Hydnum_orientalbidum
LC377874_Hydnum_albidum
LC377877_Hydnum_albidum
LC621850_Hydnum_orientalbidum
LC578779_Hydnum_albidum
LC578777_Hydnum_albidum
OR211381_Hydnum_orientalbidum
LC621861_Hydnum_orientalbidum
LC621855_Hydnum_orientalbidum
PQ505059_Hydnum_orientalbidum
LC621849_Hydnum_orientalbidum
LC621858_Hydnum_orientalbidum
OR211376_Hydnum_orientalbidum
LC578780_Hydnum_albidum
LC621854_Hydnum_orientalbidum
LC377876_Hydnum_albidum
LC621857_Hydnum_orientalbidum
LC621852_Hydnum_orientalbidum
LC621862_Hydnum_orientalbidum
OR947111_Hydnum_orientalbidum
KU612610_Hydnum_sp
MZ221555_Hydnum_repandum
MZ622031_Hydnum_albidum
OR947108_Hydnum_orientalbidum
PP455007_Hydnum_albidum
MZ310495_Hydnum_repandum
OR211375_Hydnum_orientalbidum
OR211377_Hydnum_orientalbidum
LC621846_Hydnum_orientalbidum
LC667091_Hydnum_sp
LC621845_Hydnum_orientalbidum
LC377875_Hydnum_albidum
LC578782_Hydnum_albidum
LC621853_Hydnum_orientalbidum
OQ656787_Hydnum_orientalbidum
OQ656789_Hydnum_orientalbidum
OQ656788_Hydnum_orientalbidum
LC578781_Hydnum_albidum
LC377870_Hydnum_albidum
KU612609_Hydnum_sp
Sistotrema_muscicola_taxon_154757_FINLAND
Sistotrema_muscicola_KHL_11721_FINLAND
97.9/99
42.4/89
97.5/100
96.4/99
69.2/81
87.3/96
100/100
91.3/100
96.8/99
100/100
98.1/100
80.4/97
100/100
